# Supplementary material for: A Multipatient Simulation Session: Evaluation of Six Simulated Patients with Different Shock Syndromes
Source: MedEdPORTAL. 2017 Jun 7;13:10591. doi: 10.15766/mep_2374-8265.10591 (PMC6354717; doi:10.15766/mep_2374-8265.10591)
Supplement: Supplementary file 1 — A. Prereading Assignment.docx B. Patient 1 Scenario.docx C. Patient 2 Scenario.docx D. Patient 3 Scenario.docx E. Patient 4 Scenario.docx F. Patient 5 Scenario.docx G. Patient 6 Scenario.docx H. Preformatted Evaluation Matrix.xlsx I. Completed Evaluation Matrix.xlsx J. Survey Instrument.docx [file mep-13-10591-s001.zip › A._Prereading_Assignment.docx]

**PREPARATION FOR THE SHOCK SIMULATION**

By the end of this simulation session, the learner will be able to:

- 1. Assign roles to team members to maximize team efficiency.
  2. Evaluate six simulated patients with different shock syndromes.
  3. Record and report key clinical and diagnostic findings for each simulated clinical encounter.
  4. Initiate at least one therapeutic intervention for each patient.
  5. Classify the type of shock in each patient based on data collected during the clinical encounters.
  6. Identify the etiology of shock, or make a presumptive diagnosis, for each patient.
  7. Predict the cardiac output, central venous pressure or pulmonary artery wedge pressure, and systemic vascular resistance for each patient.
  8. Explain the physiologic and pharmacologic effects of the chosen therapies for each patient.
  9. Compare the clinical findings of each of the four classes of shock.

During this 2-hour simulation exercise, you will be a member of a “Shock Response Team” that rapidly evaluates and treats patients suffering from various types of shock in a hospital setting. Your team will consist of 4-5 fellow students and one nurse. You will have 5 minutes to evaluate each patient, order tests, record key clinical findings in a chart, and attempt at least one therapeutic intervention before moving to the next patient. There is a rumor that there may be six patients who will require your services during your shift. Since this will be a very busy shift, your team should maximize efficiency by assigning roles. Choose one team leader and one recorder; supporting team members may assume other, varying responsibilities. It is best if you rotate the role assignments among your team members for each case. You will have just enough time to perform a focused history and physical. The preformatted chart will guide your efforts.

Order any laboratory, radiographic, or ultrasound test you think is appropriate. Although time will be “compressed”, some tests may not be available during the scenario. Nevertheless, you will have enough information to make decisions. A variety of medications and blood products are available. However, consultants will not be accessible. (That should not be a problem, since you are the “shock experts”.)

All three teams will quickly assemble in the Classroom to discuss the cases. During the Discussion Session, you will have 5 minutes to finalize each patient chart, classify the type of shock, and make a presumptive diagnosis. Teams will take turns reporting their key findings to the group and recording them on the projected shock evaluation matrix. Each team will also explain the physiologic and pharmacologic effects of their chosen therapies. Other teams may challenge their observations, decisions, or conclusions (10 minutes per case). There will be 36 minutes allocated to the scenarios and 70 minutes to the discussion.

To prepare for this event, you should complete the following pre-reading assignments:

1. The clinical and hemodynamic characteristics of each of the classes of shock (See Critical Care Emergency Medicine. Section XI: Special Considerations; Chapter 46: Classification of Shock

http://accessemergencymedicine.mhmedical.com.ezproxy.med.wmich.edu/content.aspx?bookid=522&sectionid=41291808);

1. Winters, ME, BeBlieux P, Marcolinie EG, et al. *Emergency Department Resuscitation of the Critically Ill*. American College of Emergency Physicians (publisher), Dallas; 2011; Chapter 1: The Patient with Undifferentiated Shock, pp. 1-4.

As we review these cases in the Discussion Session, compare the clinical findings of each of the four classes (cardiogenic, obstructive, hypovolemic, and distributive) so that you can quickly recognize the various “patterns” of shock.
