# Supplementary material for: A Multipatient Simulation Session: Evaluation of Six Simulated Patients with Different Shock Syndromes
Source: MedEdPORTAL. 2017 Jun 7;13:10591. doi: 10.15766/mep_2374-8265.10591 (PMC6354717; doi:10.15766/mep_2374-8265.10591)
Supplement: Supplementary file 1 — A. Prereading Assignment.docx B. Patient 1 Scenario.docx C. Patient 2 Scenario.docx D. Patient 3 Scenario.docx E. Patient 4 Scenario.docx F. Patient 5 Scenario.docx G. Patient 6 Scenario.docx H. Preformatted Evaluation Matrix.xlsx I. Completed Evaluation Matrix.xlsx J. Survey Instrument.docx [file mep-13-10591-s001.zip › B._Fill-In_Answer_Packet_Cases_with_Drawing.docx]

**Fill-In Answer Packet – Cases with Drawing Version**

Practice Drawing #1

Practice Drawing #2

Case 1 - Drawing

Case 1 - Answers

1. __________________________________

2.

a. __________________________________

b. __________________________________

1. __________________________________

4.

a. __________________________________

b. __________________________________

1. __________________________________

6.

a. __________________________________

b. __________________________________

1. __________________________________
2. __________________________________

Case 2 - Drawing


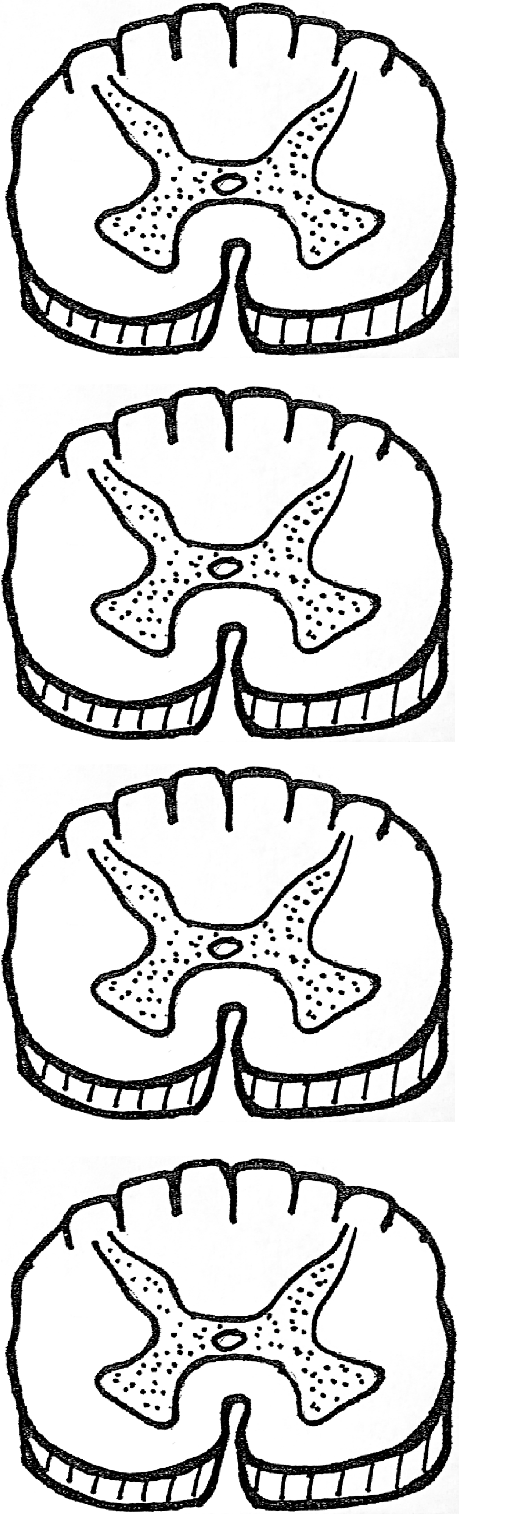


Case 2 – Answers

1. __________________________________

2.

a. __________________________________

b. __________________________________

1. __________________________________

4.

a. __________________________________

b. __________________________________

1. __________________________________

6.

a. __________________________________

b. __________________________________

1. __________________________________
2. __________________________________

Case 3 - Drawing


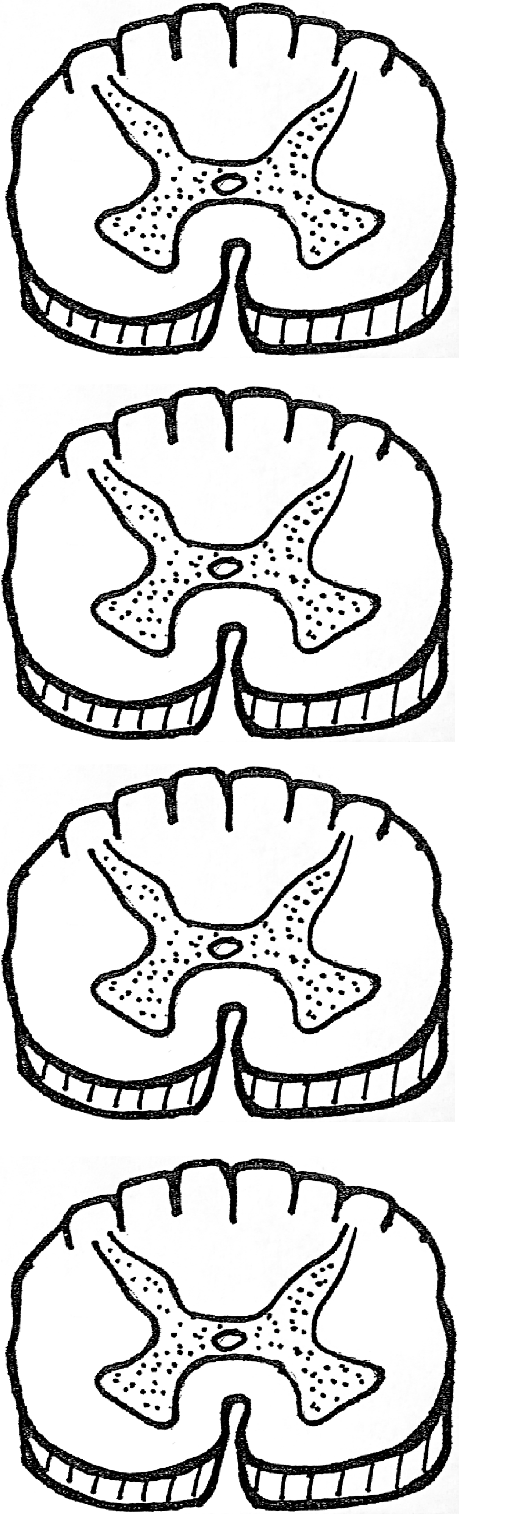


Case 3 – Answers

1. __________________________________

2.

a. __________________________________

b. __________________________________

1. __________________________________

4.

a. __________________________________

b. __________________________________

1. __________________________________

6.

a. __________________________________

b. __________________________________

1. __________________________________
2. __________________________________

Bonus Question Answers

Case 2 _____________________________

Case 3 _____________________________
