# Supplementary material for: A Multipatient Simulation Session: Evaluation of Six Simulated Patients with Different Shock Syndromes
Source: MedEdPORTAL. 2017 Jun 7;13:10591. doi: 10.15766/mep_2374-8265.10591 (PMC6354717; doi:10.15766/mep_2374-8265.10591)

| Appendix B: MedEdPORTAL Simulation Case  SIMULATION CASE TITLE: Multi-Patient Simulation Session: Evaluation of Six Simulated Patients with Different Shock Syndromes.  AUTHORS: Richard Lammers, MD, Philip Pazderka, MD, Maria Sheakley, PhD. | |
| --- | --- |
| PATIENT NAME: Jane Sweet  PATIENT AGE: 34  CHIEF COMPLAINT: Vomiting for 3 days | |
|  | |
| Brief narrative description of case | The patient is a 34-year-old diabetic female with vomiting for three days. Student teams have been informed that they are members of a shock response team, and have eight minutes to evaluate the patient, record key clinical findings in a chart, view test results, and attempt a therapeutic intervention. |
| Primary Learning Objectives | By the end of this simulation session, the learner will be able to:   1. Assign roles to each team member to maximize team efficiency. 2. Evaluate the patient and record key clinical and diagnostic findings. 3. Initiate at least one therapeutic intervention. 4. Classify the type of shock based on data collected during the clinical encounters. 5. Identify the etiology of shock, or make a presumptive diagnosis. 6. Predict cardiac output, central venous pressure, and systemic vascular resistance. 7. Explain the physiologic and pharmacologic effects of the chosen therapy. |
| Critical Actions | 1. Assign roles to each team member before entering the patient room, ensuring that someone is assigned the role of scribe and another serves as team leader. 2. Utilize the shock evaluation matrix to complete a focused history and physical exam. 3. Identify clinical findings consistent with hypovolemic shock, including sustained vomiting, hypotension, tachycardia, dry mucous membranes, weak pulses, and low jugular venous pulse (JVP). 4. Determine that the patient is in hypovolemic shock. 5. Order IV normal saline solution as treatment. |
| Learner Preparation | To prepare for this event, students should complete the following pre-reading assignments:   1. The clinical and hemodynamic characteristics of each of the classes of shock (See Critical Care Emergency Medicine. Section XI: Special Considerations; Chapter 46: Classification of Shock). 2. Winters, ME, BeBlieux P, Marcolinie EG, et al. *Emergency Department Resuscitation of the Critically Ill*. American College of Emergency Physicians (publisher), Dallas; 2011; Chapter 1: The Patient with Undifferentiated Shock, pp. 1-4. |

| INITIAL PRESENTATION | | | |
| --- | --- | --- | --- |
| Initial vital signs | Temp: 37.5^o^ C  Pulse: 130 /minute  Blood pressure: 89/40 mm Hg  Respirations: 34/minute  Oxygen saturation: 99%  Mean Arterial Pressure (MAP): 56 mm Hg | | |
| Overall Appearance | When the learners enter the room, there is a young, adult female who is wearing a hospital gown, sitting at a 60-degree angle. A pulse oximeter probe has been placed on a finger, and a nasal cannula is in place; oxygen flow at 2 L/min. The vital signs monitor has been turned on. Peripheral IV access has been established. The same array of treatment options for all cases in this simulation exercise are visible on a cart, including vasopressors, an antihistamine, an antiarrhythmic, calcium and calcium channel blocker, and steroid drugs; IV fluids and blood products; airway equipment; a defibrillator; an 18-gauge angiocath needle; and a glucose measurement device. There is also a preformatted evaluation matrix on a clipboard. | | |
| Actors and roles in the room at case start | A nurse at the bedside introduces the patient, hands an ED Triage Note to the team (see below in HPI section), and awaits instructions. During the scenario, the nurse provides further scripted information, diagnostic test results, and requested equipment. The nurse will describe physical findings that cannot be portrayed by the mannequin while staying in role. The nurse performs only those interventions requested by the learners. The nurse troubleshoots equipment and attempts to mitigate simulation artifacts that interfere with the case. The nurse receives instructions through an earpiece from an instructor in the Control Room, as needed. A simulation technician or other health care provider with basic medical knowledge (eg. EMT level) and who is familiar with the capabilities of the mannequin can play this role.  Nurse’s Initial Script:   - Hello, I’m nurse __________. - This patient just arrived, are you the Shock Team? - Her blood pressure is low and she is still vomiting. - Here is your chart, I placed an IV already*.* - Just let me know what you want me to do.   A faculty instructor is present in the Control Room. This person serves as the voice of the patient, operates the computer by triggering manual changes as scripted, guides the nurse/actor by direct-talk two-way radio, and terminates the scenario at eight minutes. The faculty instructor observes the performance of the team, provides feedback, and facilitates the debriefing/discussion session. | | |
| HPI | Information in ED Triage Note:  Patient name: Jane Sweet  Demographics: 34 y/o; female  ED arrival information: EMS  Chief complaint: Vomiting x 3 days  Significant history/details: non-adherent to medications; out of insulin x 1 week; didn’t fill prescription because unable to find transportation to the pharmacy.  Allergies: NKDA  Home medications: Lantus, Novolog insulin  Medical history: Diabetes mellitus, type 1  Surgical history: none  Social history: no smoking  Family comments: none present  Vital signs:  T: 37.5^o^C  P: 130/min  BP: 89/40  R: 34/min  O_2_ sat: 99%  Nurse’s Evaluation: Looks dry  Treatment initiated: IV access; nasal oxygen  Information volunteered by patient: Primary symptoms (vomiting for 3 days and lethargy).  Information provided by patient, if requested:  Lethargic and vomiting for 3 days. Is a type I diabetic and has been out of insulin for 1 week. | | |
| Past Medical/Surgical History | Medications | Allergies | Family History |
| Diabetes mellitus type 1; no surgical history. | Lantus, Novolog | No known medical allergies | Not known |
| Physical Examination | | | |
| General | lethargic | | |
| HEENT | dry oral mucous membranes | | |
| Neck | no jugular venous distention; trachea midline | | |
| Lungs | tachypnea; clear to auscultation | | |
| Cardiovascular | tachycardia | | |
| Abdomen | non-tender | | |
| Neurological | normal | | |
| Skin | dry; decreased turgor; pale | | |
| GU | normal | | |
| Psychiatric | lethargic; easily arousable to gentle stimulation; answers questions appropriately, though slightly slowed mentation; oriented to person, place, and time; cognition intact | | |

Diagnostic studies that are provided immediately, if ordered:

Complete blood count Normal Ranges:

White blood cells: 14.0 x 10^9^ cells/mcL (3.5-10.5 x 10^9^ cells/mcL)

Hemoglobin: 13.0 g/dL (13.5-17.5 g/dL)

Hematocrit: 38.5% (38.8-50%)

Platelets: 236,000 x10^3^ mcL (150-450 x10^3^ mcL)

Basic metabolic panel Normal Ranges:

Na (sodium): 119 mEq/L (135-144 mEq/L)

K (potassium): 5.9 mEq/L ( 3.7-5.2 mEq/L)

Cl (chloride): 84 mEq/L (97-108 mEq/L)

CO2 (bicarbonate): 5 mEq/L (22-29 mEq/L)

BUN (blood urea nitrogen): 39 mg/dL (7-20 mg/dL)

Cr (creatinine): 2.2 mg/dL (0.8-1.4 mg/dL)

Glucose: 814 mg/dL (64-128 mg/dL)

Ca (calcium) 8.7 mg/dL (8.5-10.6 mg/dL)

Lactic acid Normal Ranges:

Lactic acid: 4.1 (mEq/L) (0.5-2.2 mEq/L)

Radiology report

Chest Radiograph (Plain Film; PA & lateral views): Normal


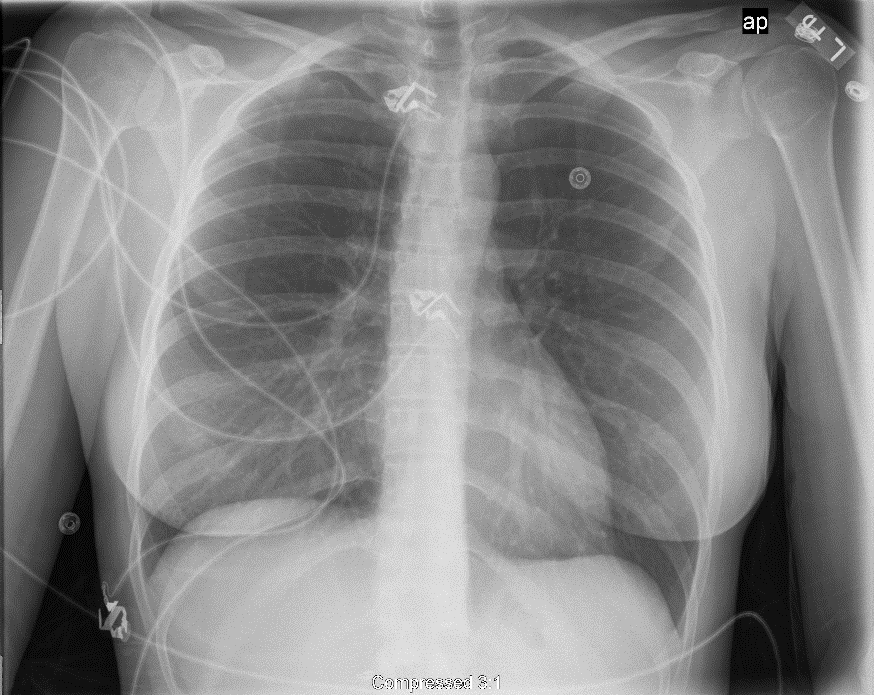


*Image from the collection of Richard Lammers, MD*


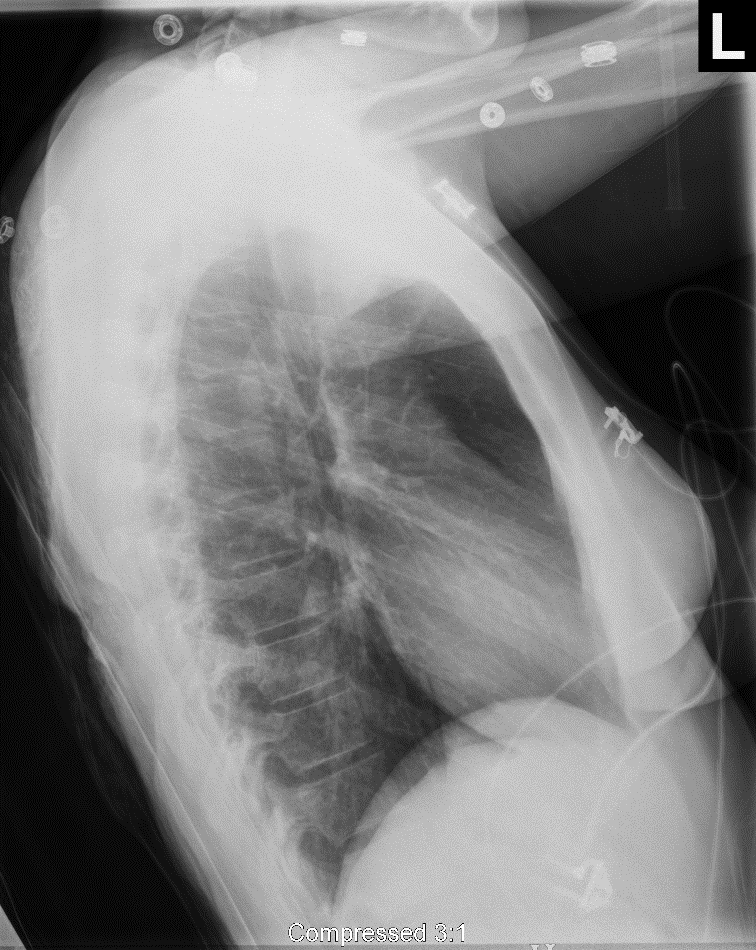


*Image from the collection of Richard Lammers, MD*

12-lead ECG

Tachycardia (130 bpm)


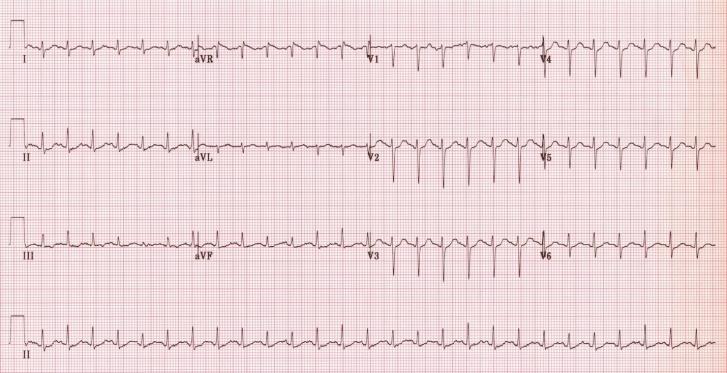


*Image from the collection of Richard Lammers, MD*

Rapid Ultrasound for Shock and Hypotension (RUSH) Examination Protocol

Subcostal Cardiac View: No pericardial effusion is present.

Apical 4 Chamber Cardiac View: Right ventricular size is normal.

Parasternal Long Axis Cardiac View: LV function is hyperdynamic.

Inferior Vena Cava View: Inferior vena cava in the short axis measures 1.5 cm. Respiratory variability is > 50%. Conclusion: volume responsive.

Right & Left Upper Quadrant Views: No intraperitoneal fluid present.

Pelvic View: No intraperitoneal fluid present in sagittal or transverse planes

Abdominal Aorta View: Aortic diameter is < 3 cm.

Thoracic View: No evidence of pneumothorax on right or left side.

| INSTRUCTOR NOTES - CHANGES AND CASE BRANCH POINTS | | |
| --- | --- | --- |
| Intervention / Time point | Change in Case | Additional Information |
| The same array of treatment options for all cases in this simulation exercise are visible on a cart. There is generally one best treatment option for each case. | | |
| *Dopamine IV drip* | *BP increases 5/5 mmHg*  *Pulse increases 10 bpm* | *Greatest affinity for dopamine receptors. Greater affinity for beta receptors than alpha receptors (D1 > B1 and B2 > a1)* |
| *Norepinephrine IV drip* | *BP increases 10/10 mmHg*  *Pulse remains unchanged* | *Greater affinity for alpha receptors than beta receptors (a1 > B1))* |
| *Epinephrine IV drip* | *BP increases 10/10 mmHg*  *Pulse increases 20 bpm* | *Greater affinity for beta receptors than alpha receptors (B1 > a1 and B2)* |
| *Epinephrine IM 0.3mg* | *BP increases 10/10 mmHg*  *Pulse increases 20 bpm* | *Greater affinity for beta receptors than alpha receptors(B1 > a1 and B2)* |
| *Phenylephrine IV drip* | *BP increases 10/10 mmHg*  *Pulse remains unchanged* | *Acts on alpha-1 receptors, no effect on beta receptors.* |
| *Benadryl 50mg IV* | *BP remains unchanged*  *Pulse remains unchanged* |  |
| *Normal Saline Bolus 1 Liter IV* | BP increases 16/20 mmHg, pulse decreases 10 bpm. | Best treatment option for this case. |
| *Needle thoracostomy* | Pulse increases 10 bpm, respirations increase 10 per minute, oxygen sat decreases 10% |  |
| *Synchronized cardioversion at 200J* | Pulse increases 10 bpm, respirations increase 5 per minute | No change in rhythm |

Ideal Scenario Flow

*Provide a detailed narrative description of the way this case should flow if participants perform in the ideal fashion.*

*The learners enter the room to find a lethargic 34-year-old female patient. The nurse informs the learners that the patient is a type I diabetic who ran out of insulin a week ago, and has been vomiting for three days. The learners immediately check the monitors and see the patient is hypotensive and tachycardic. After completing a focused history and physical examination, the learners note that the patient’s mucus membranes are dry, pulses are weak, neck veins are not visible (at 60 degrees), and respiratory rate is elevated. The learners recognize that the patient is dehydrated from the vomiting, and order IV normal saline solution to be administered. In response, the patient’s blood pressure increases and heart rate decreases. The learners are not expected to treat the underlying hyperglycemia, only the shock syndrome.*

Anticipated Management Mistakes

*Provide a list of management errors or difficulties that are commonly encountered when using this simulation case.*

*Failure to assign roles: Medical student teams may not assign roles or divide tasks among themselves, resulting in inefficiencies, or repeating some tasks while ignoring others.*

*Difficulty predicting the patients cardiac output, central venous pressure, and systemic vascular resistance: During the debriefing session, the teams are asked to predict the patient’s CO, CVP, and SVR, based on the type of shock. A facilitator is sometimes required to walk the groups through this thought process (i.e. MAP = CO x SVR) for the first 1-2 patients. The teams are typically able to figure this out on their own for the remaining patients.*

*Diagnosis of underlying etiology: The teams are asked to determine the class of shock and underlying etiology of shock for each patient. 12/12 teams correctly diagnosed this as hypovolemic shock, but only 9/12 correctly diagnosed the underlying etiology of diabetic ketoacidosis. All three teams who missed the etiology did not attempt a diagnosis (the box was blank).*

Completed shock evaluation matrix for Jane Sweet:


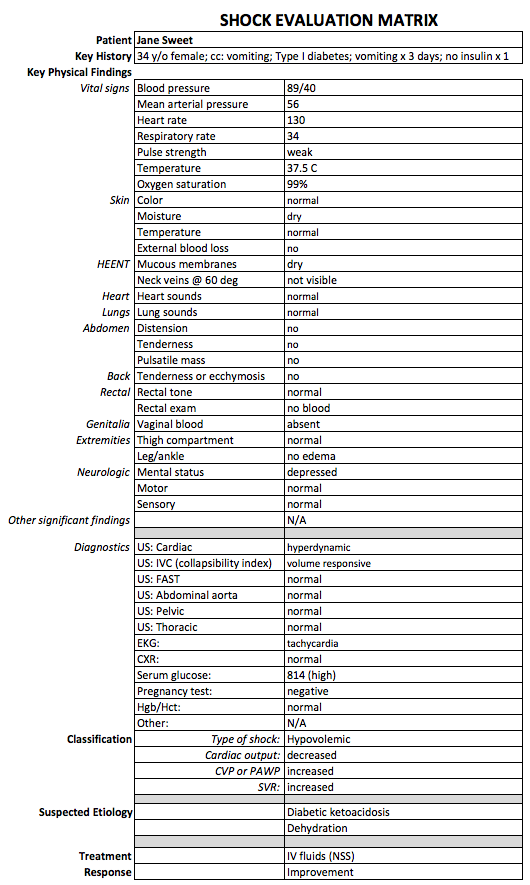

Supplement: Supplementary file 1 — A. Prereading Assignment.docx B. Patient 1 Scenario.docx C. Patient 2 Scenario.docx D. Patient 3 Scenario.docx E. Patient 4 Scenario.docx F. Patient 5 Scenario.docx G. Patient 6 Scenario.docx H. Preformatted Evaluation Matrix.xlsx I. Completed Evaluation Matrix.xlsx J. Survey Instrument.docx [file mep-13-10591-s001.zip › B._Patient_1_Scenario.docx]
