# Supplementary material for: A Multipatient Simulation Session: Evaluation of Six Simulated Patients with Different Shock Syndromes
Source: MedEdPORTAL. 2017 Jun 7;13:10591. doi: 10.15766/mep_2374-8265.10591 (PMC6354717; doi:10.15766/mep_2374-8265.10591)

| Appendix C: MedEdPORTAL Simulation Case  SIMULATION CASE TITLE: Multi-Patient Simulation Session: Evaluation of Six Simulated Patients with Different Shock Syndromes.  AUTHORS: Richard Lammers, MD, Philip Pazderka, MD, Maria Sheakley, PhD. | |
| --- | --- |
| PATIENT NAME: John Swift  PATIENT AGE: 76  CHIEF COMPLAINT: Shortness of breath and palpitations | |
|  | |
| Brief narrative description of case | This patient is a 76-year-old male with sudden onset of shortness of breath and palpitations. He was brought to the emergency department by EMS. Student teams have been informed that they are members of a shock response team, and have eight minutes to evaluate the patient, record key clinical findings in a chart, view test results, and attempt a therapeutic intervention. |
| Primary Learning Objectives | By the end of this simulation session, the learner will be able to:   1. Assign roles to each team member to maximize team efficiency. 2. Evaluate the patient and record key clinical and diagnostic findings. 3. Initiate at least one therapeutic intervention. 4. Classify the type of shock based on data collected during the clinical encounters. 5. Identify the etiology of shock, or make a presumptive diagnosis. 6. Predict cardiac output, central venous pressure, and systemic vascular resistance. 7. Explain the physiologic and pharmacologic effects of the chosen therapy. |
| Critical Actions | 1. Assign roles to each team member before entering the patient room, ensuring that someone is assigned the role of scribe and another serves as team leader. 2. Utilize the shock evaluation matrix to complete a focused history and physical exam. 3. Order an ECG and correctly interpret the arrhythmia as atrial fibrillation with rapid ventricular response. 4. Identify clinical findings consistent with rate-related cardiogenic shock secondary to atrial fibrillation with RVR, including hypotension, tachycardia, weak pulses, high respiratory rate and rales, cool and pale skin, and diaphoresis. 5. Determine that the patient is in cardiogenic due to atrial fibrillation. 6. Perform cardioversion to restore a normal rhythm. |
| Learner Preparation | To prepare for this event, students should complete the following pre-reading assignments:   1. The clinical and hemodynamic characteristics of each of the classes of shock (See Critical Care Emergency Medicine. Section XI: Special Considerations; Chapter 46: Classification of Shock). 2. Winters, ME, BeBlieux P, Marcolinie EG, et al. *Emergency Department Resuscitation of the Critically Ill*. American College of Emergency Physicians (publisher), Dallas; 2011; Chapter 1: The Patient with Undifferentiated Shock, pp. 1-4. |

| INITIAL PRESENTATION | | | |
| --- | --- | --- | --- |
| Initial vital signs | Temp: 37.5^o^ C  Pulse: 160 /minute  Blood pressure: 70/50 mm Hg  Respirations: 30 /minute  Oxygen saturation: 89%  Mean Arterial Pressure (MAP): 57 mm Hg | | |
| Overall Appearance  *What do learners see when they first enter the room?* | When the learners enter the room, there is an adult male who is wearing a hospital gown, sitting at a 60-degree angle. He is short of breath, pale, and diaphoretic. A pulse oximeter probe has been placed on a finger, and he is wearing a non-rebreather mask; oxygen flow at 12 L/min. The vital signs monitor has been turned on. Peripheral IV access has been established and a 1-liter bag of normal saline has been started at a ‘KVO’ (keep vein open) rate. The same array of treatment options for all cases in this exercise are visible on a cart, including vasopressors, an antihistamine, an antiarrhythmic, calcium and calcium channel blocker, and steroid drugs; IV fluids and blood products; airway equipment; a defibrillator; an 18-gauge angiocath needle; and a glucose measurement device. | | |
| Actors and roles in the room at case start  *Who is present at the beginning and what is their role? Who may play them?* | A nurse at the bedside introduces the patient, hands an ED Triage Note to the team (see below in HPI section), and awaits instructions. During the scenario, the nurse provides further scripted information, diagnostic test results, and requested equipment. The nurse will describe physical findings that cannot be portrayed by the mannequin while staying in role. The nurse performs only those interventions requested by the learners. The nurse troubleshoots equipment and attempts to mitigate simulation artifacts that interfere with the case. The nurse receives instructions through an earpiece from an instructor in the Control Room, as needed. A simulation technician or other health care provider with basic medical knowledge (eg. EMT level) and who is familiar with the capabilities of the mannequin can play this role.  Nurse’s Introduction Script:   - Hello, I’m nurse __________, are you the Shock Team? - This patient was brought in by paramedics. - He is short of breath. - Here is your chart. - I started an IV with normal saline, what rate would you like me to run it?   A faculty instructor is present in the Control Room. This person serves as the voice of the patient, operates the computer by triggering manual changes as scripted, guides the nurse/actor by direct-talk two-way radio, and terminates the scenario at eight minutes. The faculty instructor observes the performance of the team, provides feedback, and facilitates the debriefing/discussion session. | | |
| HPI | Information in ED Triage Note:  Patient name: John Swift  Demographics: 76 y/o male  ED arrival information: EMS  Chief complaint: shortness of breath x 1 hour; palpitations  Significant history/details: sudden onset  Allergies: NKDA  Home medications: aspirin  Medical history: coronary artery disease  Surgical history: none  Social history: no smoking  Family comments: none present  Vital signs:  T: 37.5^o^C  P: 160/min  BP: 70/50  R: 30/min  O_2_ sat: 89%  Nurse’s Evaluation: Priority 1—hypotensive  Treatment initiated: IV line; oxygen by non-rebreathing mask, 12 L/min  Information volunteered by patient: Primary symptoms (shortness of breath and palpitations)  Information provided by patient, if requested: Short of breath. Feels like heart is beating out of chest. Feeling anxious. | | |
| Past Medical/Surgical History | Medications | Allergies | Family History |
| Coronary artery disease; no surgical history | Aspirin | No known allergies | Not known |
| Physical Examination | | | |
| General | awake and anxious-appearing | | |
| HEENT | moist oral mucous membranes | | |
| Neck | no jugular venous distention; trachea midline | | |
| Lungs | tachypnea, bilateral rales | | |
| Cardiovascular | tachycardia; irregularly irregular rhythm | | |
| Abdomen | non-tender | | |
| Neurological | normal | | |
| Skin | pale; cool; diaphoretic; normal turgor | | |
| GU | normal | | |
| Psychiatric | awake; oriented to person, place, and time; anxious; cognition intact | | |

Diagnostic studies that are provided immediately if ordered:

Complete blood count Normal Ranges:

WBC’s: 12.5 x 10^9^ cells/mcL (3.5-10.5 x 10^9^ cells/mcL)

Hemoglobin: 16.0 g/dL (13.5-17.5 g/dL)

Hematocrit: 47.8% (38.8-50%)

Platelets: 400,000 x10^3^ mcL (150-450 x10^3^ mcL)

Basic metabolic panel Normal Ranges:

Na (sodium): 140 mEq/L (135-144 mEq/L)

K (potassium): 5.0 mEq/L (3.7-5.2 mEq/L)

Cl (chloride): 97 mEq/L (97-108 mEq/L)

CO2 (bicarbonate): 25 mEq/L (22-29 mEq/L)

BUN (blood urea nitrogen): 25 mg/dL (7-20 mg/dL)

Cr (creatinine): 1.5 mg/dL (0.8-1.4 mg/dL)

Glucose: 110 mg/dL (64-128 mg/dL)

Ca (calcium) 8.7 mg/dL (8.5-10.6 mg/dL)

Lactic Acid Normal Ranges

Lactic acid: 4.1 (mEq/L) (0.5-2.2 mEq/L)

Radiology Report

Chest Radiograph (Plain Film; AP view):

Normal heart size; bilateral pulmonary vascular congestion; normal mediastinal and hilar structures; normal bones and soft tissues. Conclusion: Findings are consistent with early pulmonary edema.

Chest film


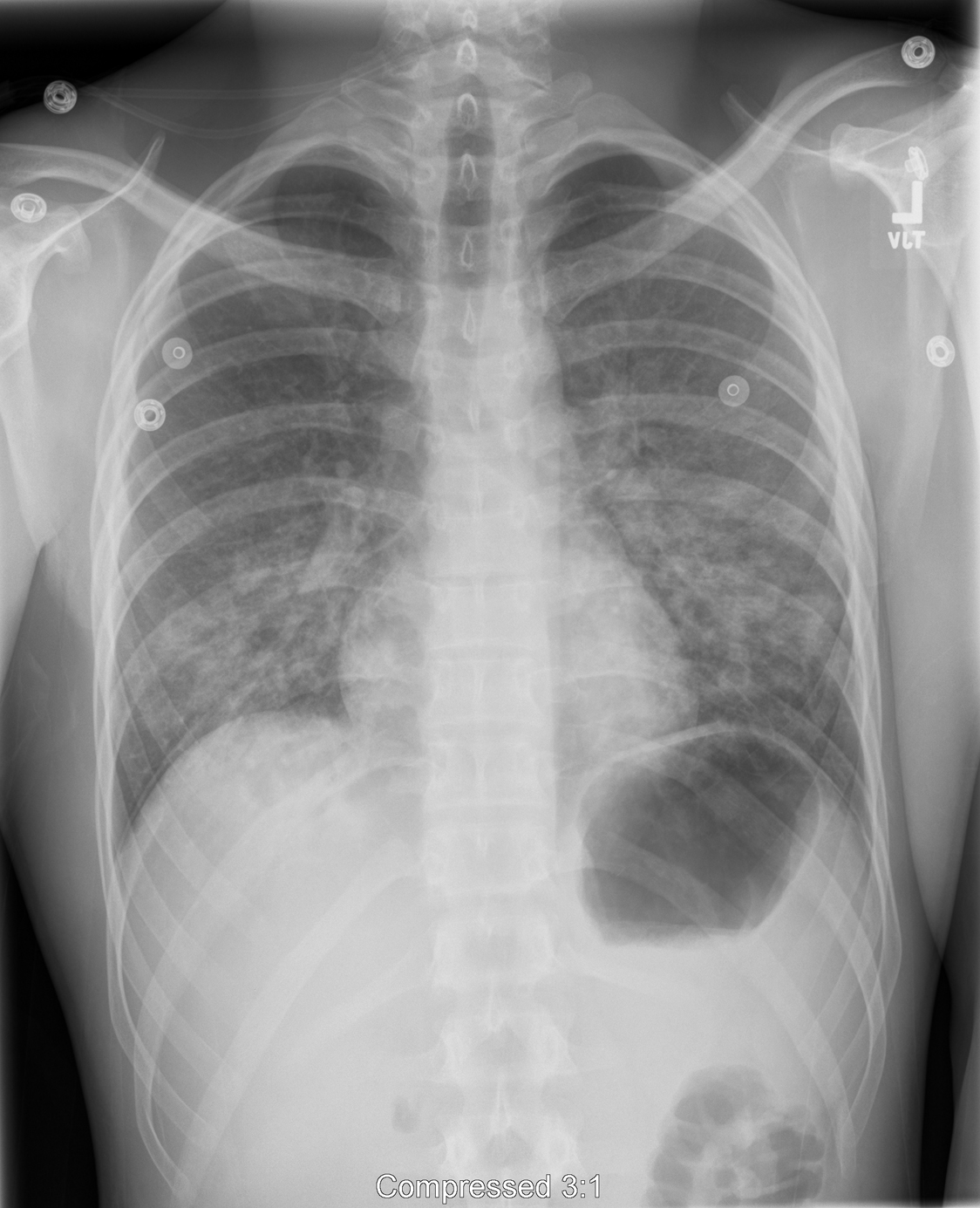


*Image from the collection of Richard Lammers, MD*


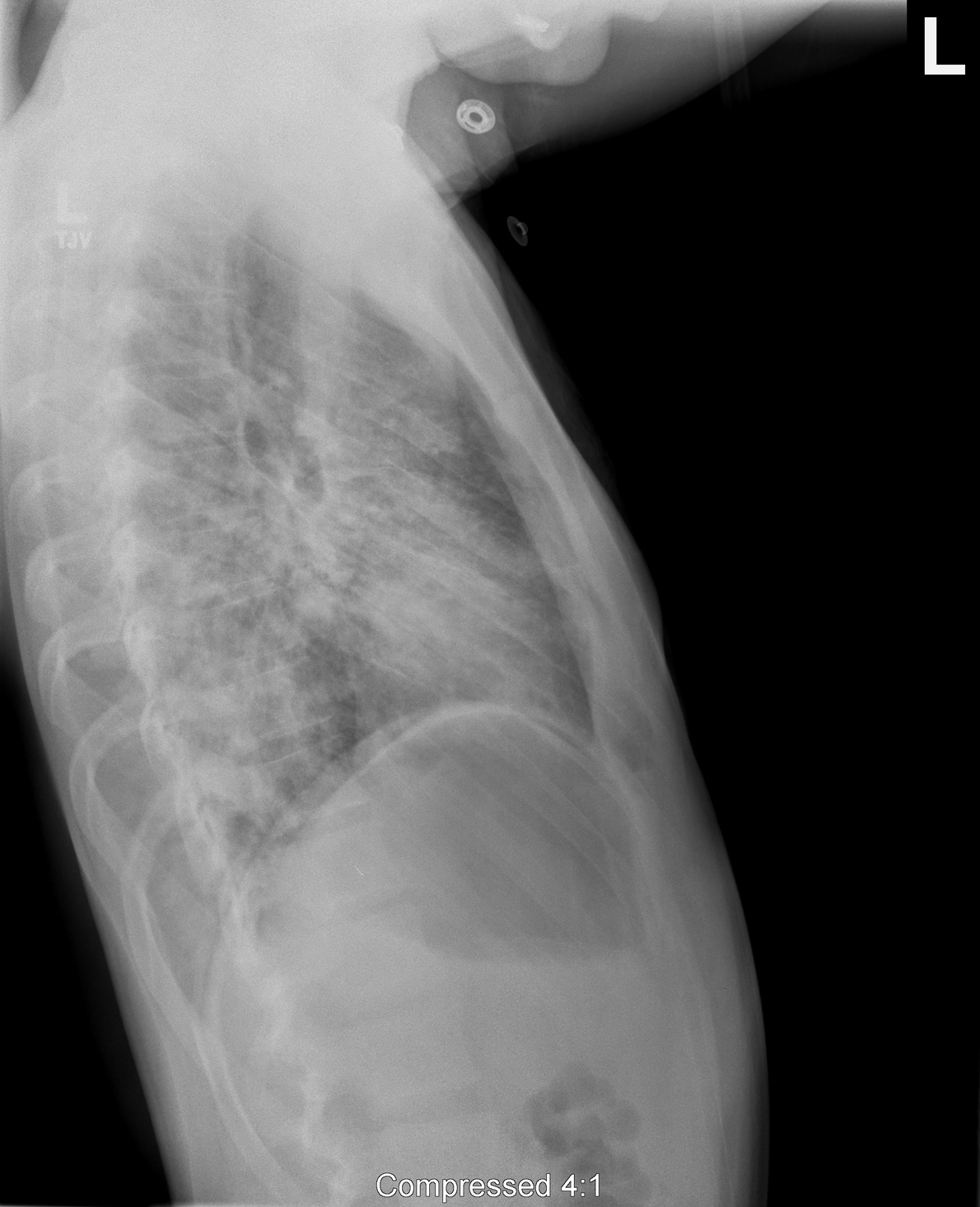


*Image from the collection of Richard Lammers, MD*

12-lead ECG:


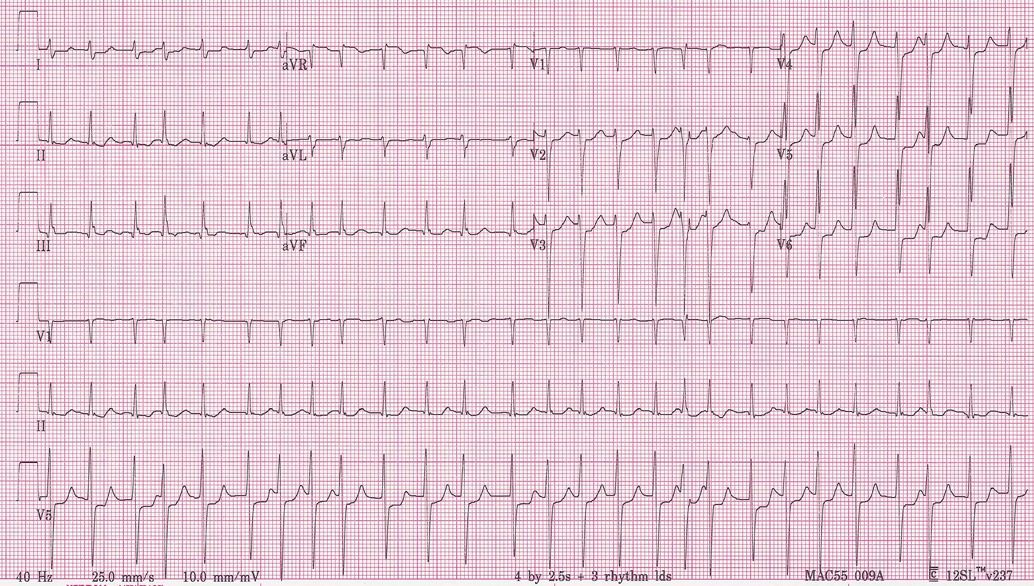


*Image from the library of Richard Lammers, MD*

Rapid Ultrasound for Shock and Hypotension (RUSH) Examination Protocol

Subcostal Cardiac View: No pericardial effusion is present.

Apical 4 Chamber Cardiac View: Right ventricular size is normal.

Parasternal Long Axis Cardiac View: LV function is hyperdynamic.

Inferior Vena Cava View: Inferior vena cava in the short axis measures 2.5 cm. Respiratory variability is <20%. Conclusion: volume unresponsive .

Right & Left Upper Quadrant Views: No intraperitoneal fluid present.

Pelvic View: No intraperitoneal fluid present in sagittal or transverse planes.

Abdominal Aorta View: Aortic diameter is < 3 cm.

Thoracic View: No evidence of pneumothorax on right or left side.

| INSTRUCTOR NOTES - CHANGES AND CASE BRANCH POINTS | | |
| --- | --- | --- |
| Intervention / Time point | Change in Case | Additional Information |
| *Dopamine IV drip* | *BP increases 5/5 mmHg*  *Pulse increases 10 bpm* | *Greatest affinity for dopamine receptors. Greater affinity for beta receptors than alpha receptors (D1 > B1 and B2 > a1)* |
| *Norepinephrine IV drip* | *BP increases 10/10 mmHg*  *Pulse remains unchanged* | *Greater affinity for alpha receptors than beta receptors (a1 > B1))* |
| *Epinephrine IV drip* | *BP increases 10/10 mmHg*  *Pulse increases 20 bpm* | *Greater affinity for beta receptors than alpha receptors (B1 > a1 and B2)* |
| *Epinephrine IM 0.3mg* | *BP increases 10/10 mmHg*  *Pulse increases 20 bpm* | *Greater affinity for beta receptors than alpha receptors(B1 > a1 and B2)* |
| *Phenylephrine IV drip* | *BP increases 10/10 mmHg*  *Pulse remains unchanged* | *Acts on alpha-1 receptors, no effect on beta receptors.* |
| *Benadryl 50mg IV* | *BP remains unchanged*  *Pulse remains unchanged* |  |
| *Normal Saline Bolus 1 Liter IV* | *BP increases 10/10 mmHg* |  |
| *Needle thoracostomy* | *BP remains unchanged*  *Pulse increases 10 bpm*  *Respirations increase 10 per minute*  *Oxygen sat decreases by 10%* |  |
| *Synchronized cardioversion at 200J* | *BP increases 45/20 mmHg*  *Pulse decreases 80 bpm*  *Respirations decrease 10 per minute*  *Oxygen sat increases by 2%* | *Correct treatment option for atrial fibrillation. Normal sinus rhythm is achieved (pulse = 87 bpm).* |

Ideal Scenario Flow

*Provide a detailed narrative description of the way this case should flow if participants perform in the ideal fashion.*

The learners enter the room to find a dyspneic and diaphoretic 76-year-old male patient. The nurse informs the learners that the patient is complaining of shortness of breath and palpitations, and that she has started a bag of IV saline and put a non-rebreather mask on the patient. The learners immediately check the monitors and see the patient is hypotensive and tachycardic. While completing a focused history the learners discover the patient has coronary artery disease. On physical exam, they discover weak pulses, elevated respiration rate, low oxygen sat, and hear rales bilaterally. They order an ECG and chest x-ray (CXR). The ECG shows an irregularly irregular rhythm (atrial fibrillation) with a rapid ventricular response. The CXR shows bilateral congestion. The learners perform cardioversion and restore a normal sinus rhythm.

Anticipated Management Mistakes

*Provide a list of management errors or difficulties that are commonly encountered when using this simulation case.*

*Difficulty predicting the patients cardiac output, central venous pressure, and systemic vascular resistance: During the debriefing session, we ask the teams to predict the patient’s CO, CVP, and SVR, based on the type of shock they are experiencing. This sometimes requires a facilitator to walk the groups through the thought process for this (i.e. MAP = CO x SVR) for the few patients. The teams are typically able to figure this out on their own for the last 3-4 patients.*

*Diagnosis of underlying etiology: The teams are asked to determine the class of shock and underlying etiology of shock for each patient. 11/12 teams correctly diagnosed this as cardiogenic shock, but 0/12 correctly diagnosed the underlying etiology of atrial fibrillation with rapid ventricular rate. During the debriefing session, it became evident that the groups had difficulty identifying atrial fibrillation on the ECG tracing, since the heart rate was so rapid. This became a learning point in the debriefing sessions.*

Completed shock evaluation matrix for John Swift:


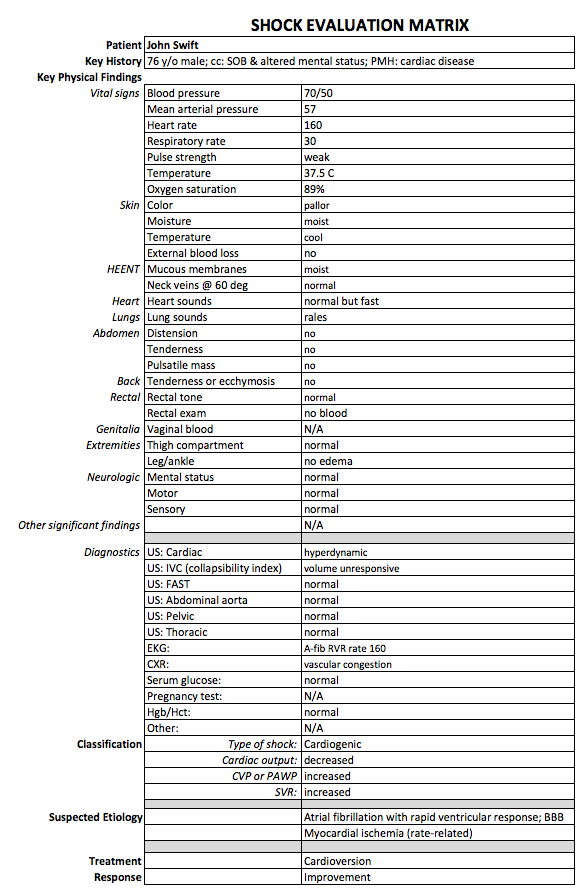

Supplement: Supplementary file 1 — A. Prereading Assignment.docx B. Patient 1 Scenario.docx C. Patient 2 Scenario.docx D. Patient 3 Scenario.docx E. Patient 4 Scenario.docx F. Patient 5 Scenario.docx G. Patient 6 Scenario.docx H. Preformatted Evaluation Matrix.xlsx I. Completed Evaluation Matrix.xlsx J. Survey Instrument.docx [file mep-13-10591-s001.zip › C._Patient_2_Scenario.docx]
