# Supplementary material for: A Multipatient Simulation Session: Evaluation of Six Simulated Patients with Different Shock Syndromes
Source: MedEdPORTAL. 2017 Jun 7;13:10591. doi: 10.15766/mep_2374-8265.10591 (PMC6354717; doi:10.15766/mep_2374-8265.10591)

| Appendix D: MedEdPORTAL Simulation Case  SIMULATION CASE TITLE: Multi-Patient Simulation Session: Evaluation of Six Simulated Patients with Different Shock Syndromes.  AUTHORS: Richard Lammers, MD, Philip Pazderka, MD, Maria Sheakley, PhD. | |
| --- | --- |
| PATIENT NAME: Pat Roach  PATIENT AGE: 29  CHIEF COMPLAINT: Chest pain and shortness of breath | |
|  | |
| Brief narrative description of case | This patient is a 29-year-old male who presents to the emergency department with acute shortness of breath. He admits that it started while he was holding his breath after inhaling marijuana. Student teams have been informed that they are members of a shock response team, and have eight minutes to evaluate the patient, record key clinical findings in a chart, view test results, and attempt a therapeutic intervention. |
| Primary Learning Objectives | By the end of this simulation session, the learner will be able to:   1. Assign roles to each team member to maximize team efficiency. 2. Evaluate the patient and record key clinical and diagnostic findings. 3. Initiate at least one therapeutic intervention. 4. Classify the type of shock based on data collected during the clinical encounter. 5. Identify the etiology of shock, or make a presumptive diagnosis. 6. Predict cardiac output, central venous pressure, and systemic vascular resistance. 7. Explain the physiologic and pharmacologic effects of the chosen therapy. |
| Critical Actions | 1. Assign roles to each team member before entering the patient room, ensuring that someone assumes the role of scribe and another serves as team leader. 2. Utilize the shock evaluation matrix to complete a focused history and physical exam. 3. Identify clinical findings consistent with tension pneumothorax, including dyspnea, hypotension and tachycardia, distended neck veins, tracheal deviation, and decreased breath sounds on the left. 4. Provide supplemental oxygen with a non-rebreather mask at a flow rate of >12L/min. 5. Determine that the patient is in obstructive shock. 6. Describe or perform a left-sided needle thoracostomy. |
| Learner Preparation | To prepare for this event, students should complete the following pre-reading assignments:   1. The clinical and hemodynamic characteristics of each of the classes of shock (See Critical Care Emergency Medicine. Section XI: Special Considerations; Chapter 46: Classification of Shock). 2. Winters, ME, DeBlieux P, Marcolinie EG, et al. *Emergency Department Resuscitation of the Critically Ill*. American College of Emergency Physicians (publisher), Dallas; 2011; Chapter 1: The Patient with Undifferentiated Shock, pp. 1-4. |

| INITIAL PRESENTATION | | | |
| --- | --- | --- | --- |
| Initial vital signs | Temp: 37.5^o^ C  Pulse: 120 /minute  Blood pressure: 80/40 mm Hg  Respirations: 34/minute  Oxygen saturation: 84%  Mean Arterial Pressure (MAP): 53 mm Hg | | |
| Overall Appearance | When the learners enter the room, there is a young, adult male who is wearing a hospital gown, sitting upright, and is in respiratory distress and diaphoretic. A pulse oximeter probe has been placed on a finger, and cardiac electrodes are properly placed. The vital signs monitor has been turned on. The patient is *not* receiving oxygen. Peripheral IV access has been established. The same array of treatment options for all cases in this exercise are visible on a cart, including vasopressors, an antihistamine, an antiarrhythmic, calcium and calcium channel blocker, and steroid drugs; IV fluids and blood products; airway equipment; a defibrillator; an 18-gauge angiocath needle; and a glucose measurement device. | | |
| Actors and roles in the room at case start | A nurse at the bedside introduces the patient, hands an ED Triage Note to the team (see below in HPI section), and awaits instructions. During the scenario, the nurse provides further scripted information, diagnostic test results, and requested equipment. The nurse will describe physical findings that cannot be portrayed by the mannequin while staying in role. The nurse performs only those interventions requested by the learners. The nurse troubleshoots equipment and attempts to mitigate simulation artifacts that interfere with the case. The nurse receives instructions through an earpiece from an instructor in the Control Room, as needed. A simulation technician or other health care provider with basic medical knowledge (eg. EMT level) and who is familiar with the capabilities of the mannequin can play this role.  Nurse’s Initial Script:   - Hello, I’m nurse ___________________ . - Are you the Shock Team? - This patient is 29-years old, and he has no cardiac disease. - He’s been hyperventilating since he was at the triage desk. - Here is the triage note and your chart. - I placed an IV already. - Do you want me to give him something for his panic attack?   A faculty instructor is present in the Control Room. This person serves as the voice of the patient, operates the computer by triggering manual changes as scripted, guides the nurse/actor by direct-talk two-way radio, and terminates the scenario at eight minutes. The faculty instructor observes the performance of the team, provides feedback, and facilitates the debriefing/discussion session. | | |
| HPI | Information in ED Triage Note:  Patient name: Pat Roach  Demographics: 29 y/o; male  ED arrival information: car  Chief complaint: Shortness of breath x 30 mins; chest pain  Significant history/details: sudden onset left-sided chest pain & SOB while holding breath  Allergies: NKDA  Home medications: none  Medical history: none  Surgical history: none  Social history: occasional marijuana  Safety screen (Feels safe at home?): yes  Family comments: none  Vital signs:  T: 37.5^o^C  P: 120/min  BP: 80/40  R: 34/min  O_2_ sat: 84%  Nurse’s Evaluation: “Appears to be anxious and in respiratory distress, but might be hyperventilating”  Treatment initiated: saline lock  Information volunteered by patient: Primary symptoms (shortness of breath and chest pain)  Information provided by patient, if requested:  Moderately severe chest pain located on left side; sudden onset while holding breath and smoking marijuana 30 minutes prior to arrival; associated with increasing shortness of breath; no trauma; no recent illnesses. | | |
| Past Medical/Surgical History | Medications | Allergies | Family History |
| None | None | No known medication allergies | Negative |
| Physical Examination | | | |
| General | *All findings are normal, except as described below:*  alert; in significant respiratory distress; speaking in 3-word sentences | | |
| HEENT | moist oral mucous membranes | | |
| Neck | distended neck veins in all positions; trachea deviated to the right | | |
| Lungs | tachypnea; clear to auscultation on the right; decreased breath sounds on the left | | |
| Cardiovascular | tachycardia | | |
| Abdomen | non-tender | | |
| Neurological | normal; no focal findings | | |
| Skin | diaphoretic; normal turgor and color; no subcutaneous emphysema | | |
| GU | normal | | |
| Psychiatric | awake, though slightly slowed mentation; oriented to person, place, and time; mood normal; cognition intact | | |

Diagnostic studies that are provided immediately, if ordered:

Complete blood count Normal Ranges:

White blood cells: 13.0 x 10^9^ cells/mcL (3.5-10.5 x 10^9^ cells/mcL)

Hemoglobin: 15.1 g/dL (13.5-17.5 g/dL)

Hematocrit: 44.9% (38.8-50%)

Platelets: 330,000 x10^3^ mcL (150-450 x10^3^ mcL)

Basic metabolic panel Normal Ranges:

Na (sodium): 140 mEq/L (135-144 mEq/L)

K (potassium): 4.0 mEq/L (3.7-5.2 mEq/L)

Cl (chloride): 97 mEq/L (97-108 mEq/L)

CO2 (bicarbonate): 25 mEq/L (22-29 mEq/L)

BUN (blood urea nitrogen): 15 mg/dL (7-20 mg/dL)

Cr (creatinine): 0.8 mg/dL (0.8-1.4 mg/dL)

Glucose: 80 mg/dL (64-128 mg/dL)

Ca (calcium) 8.7 mg/dL (8.5-10.6 mg/dL)

Lactic Acid Normal Ranges:

Lactic acid: 3.0 (mEq/L) (0.5-2.2 mEq/L)

Radiology report:

Chest Radiograph (Plain Film; AP view):

Heart: normal size

Lungs: left-sided pneumothorax with 100% lung collapse; no infiltrates or effusions

Mediastinum & hilar structures: midline structures are shifted to the right

Bones: normal

Soft tissues: normal; no subcutaneous emphysema

Conclusion: Left tension pneumothorax


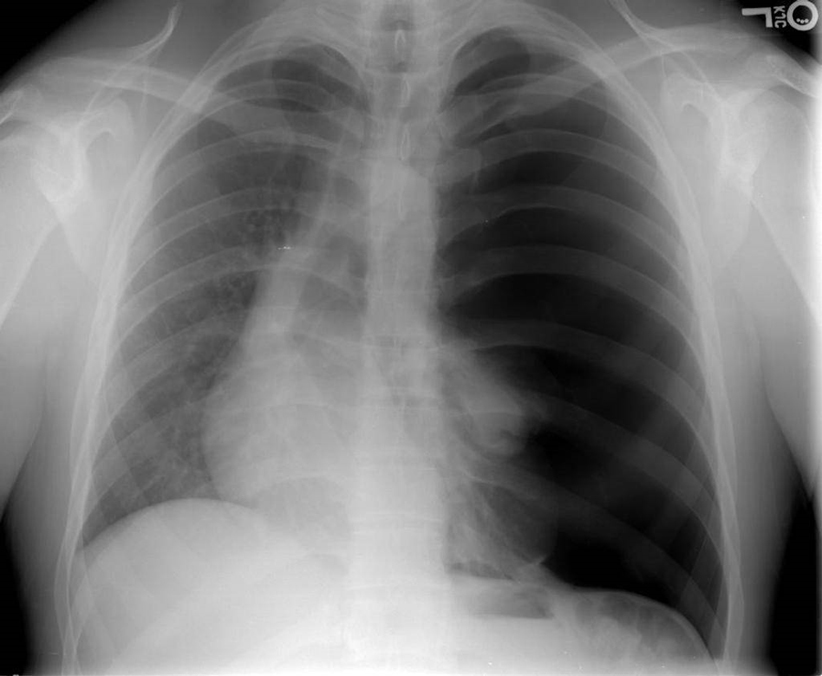


*Image from the collection of Richard Lammers, MD*

12-lead ECG:


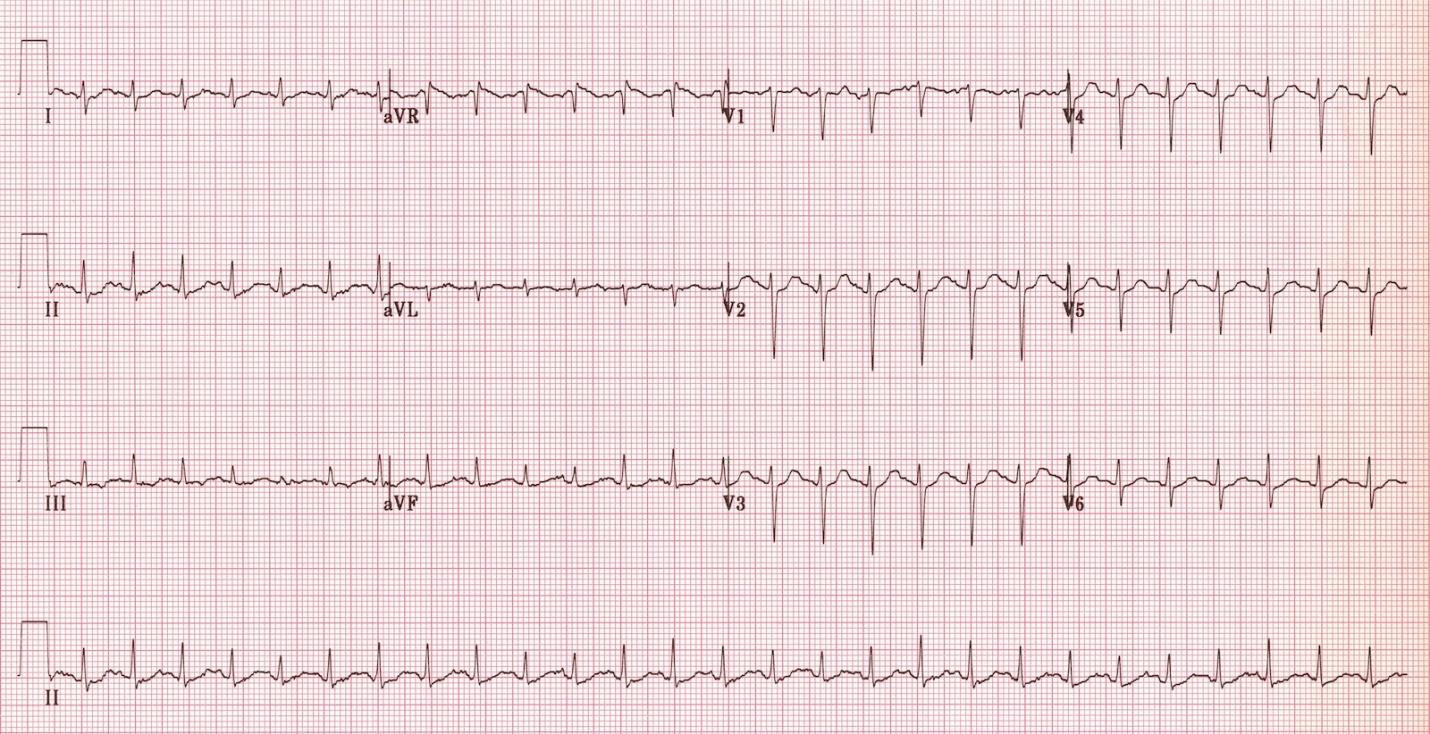


*Image from the collection of Richard Lammers, MD*

Rapid Ultrasound for Shock and Hypotension (RUSH) Examination Protocol

Subcostal Cardiac View: No pericardial effusion is present.

Apical 4 Chambered Cardiac View: Right ventricular size is normal.

Parasternal Long Axis Cardiac View: LV function is hyperdynamic

Inferior Vena Cava View: Inferior vena cava in the short axis measures 2.0 cm. Respiratory variability = 50%. Conclusion: normal.

Right & Left Upper Quadrant Views: No intraperitoneal fluid present.

Pelvic View: No intraperitoneal fluid present in sagittal or transverse planes.

Abdominal Aorta View: Aortic diameter is < 3 cm.

Thoracic View: Evidence of pneumothorax on left side.

| INSTRUCTOR NOTES - CHANGES AND CASE BRANCH POINTS | | |
| --- | --- | --- |
| Intervention / Time point | Change in Case | Additional Information |
| The same array of treatment options for all cases in this simulation exercise are visible on a cart. There is generally one best treatment option for each case. | | |
| *Dopamine IV drip* | *BP increases 5/5 mmHg*  *Pulse increases 10 bpm* | *Greatest affinity for dopamine receptors. Greater affinity for beta receptors than alpha receptors (D1 > B1 and B2 > a1)* |
| *Norepinephrine IV drip* | *BP increases 10/10 mmHg*  *Pulse remains unchanged* | *Greater affinity for alpha receptors than beta receptors (a1 > B1))* |
| *Epinephrine IV drip* | *BP increases 10/10 mmHg*  *Pulse increases 20 bpm* | *Greater affinity for beta receptors than alpha receptors (B1 > a1 and B2)* |
| *Epinephrine IM 0.3mg* | *BP increases 10/10 mmHg*  *Pulse increases 20 bpm* | *Greater affinity for beta receptors than alpha receptors(B1 > a1 and B2)* |
| *Phenylephrine IV drip* | *BP increases 10/10 mmHg*  *Pulse remains unchanged* | *Acts on alpha-1 receptors, no effect on beta receptors.* |
| *Benadryl 50mg IV* | *BP remains unchanged*  *Pulse remains unchanged* |  |
| *Normal Saline Bolus 1 Liter IV* | BP increases 10/10 mm Hg |  |
| *Needle thoracostomy* | BP increases 30/25 mm Hg  Pulse decreases 30/min  Respirations decrease 15/min  Oxygen saturation increases 12% | Best treatment option for this case. Patient describes relief of dyspnea and chest pain. |
| *Synchronized cardioversion at 200J* | Respirations increase 5/min | No change in rhythm |

Ideal Scenario Flow

*Provide a detailed narrative description of the way this case should flow if participants perform in the ideal fashion.*

*The learners enter the room to find a patient in respiratory distress. They assign team roles and should immediately review the bedside monitors and recognize that the patient is hypoxic and hypotensive. The team leader assigns roles to each team member, if not done previously. Supplemental oxygen with a non-rebreather mask and high flow rate is provided and an IV fluid bolus is ordered. After obtaining an abbreviated but appropriate history and completing a focused physical examination, the learners identify absent breath sounds on the left side and other findings consistent with a tension pneumothorax. The patient’s respiratory distress gradually worsens during the evaluation, and IV fluids provide minimal improvement in blood pressure. One member of the team performs a left-sided needle thoracostomy, audibly decompressing the pneumothorax. Blood pressure, oxygen saturation, and pulse normalize, in that sequence. The team prepares to insert a chest tube or other thoracostomy device prior to diagnostic studies. The team should complete these tasks within eight minutes.*

Anticipated Management Mistakes

*Provide a list of management errors or difficulties that are commonly encountered when using this simulation case.*

*Failure to assign roles: Medical student teams may not assign roles or divide tasks among themselves, resulting in inefficiencies, or repeating some tasks while ignoring others.*

*Failure to recognize the pneumothorax: Some students either fail to perform an appropriate exam or are not confident in their physical findings, leading to delay in diagnosis. If the mannequin’s (or a patient’s) chest is auscultated close to the midline, the difference in breath sounds may not be appreciated. Most teams order a bedside chest radiograph to confirm the diagnosis. Some teams do not recall the treatment for this emergency.*

Completed shock evaluation matrix for Pat Roach:


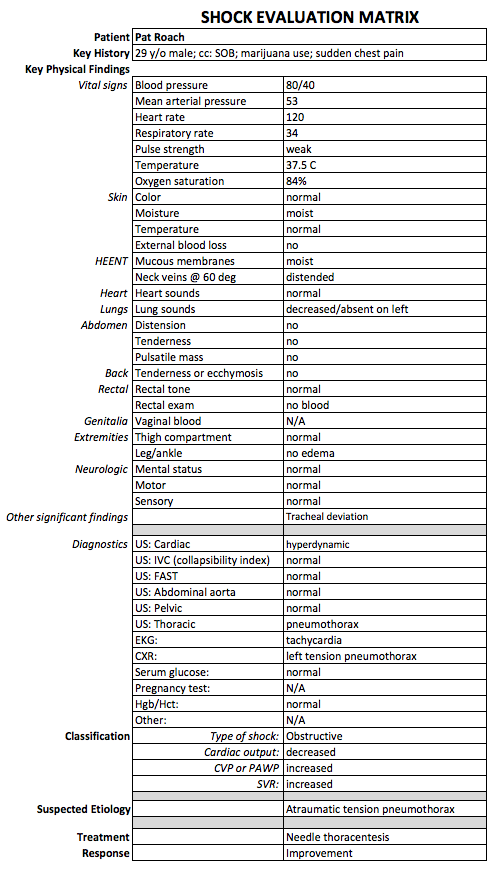

Supplement: Supplementary file 1 — A. Prereading Assignment.docx B. Patient 1 Scenario.docx C. Patient 2 Scenario.docx D. Patient 3 Scenario.docx E. Patient 4 Scenario.docx F. Patient 5 Scenario.docx G. Patient 6 Scenario.docx H. Preformatted Evaluation Matrix.xlsx I. Completed Evaluation Matrix.xlsx J. Survey Instrument.docx [file mep-13-10591-s001.zip › D._Patient_3_Scenario.docx]
