# Supplementary material for: A Multipatient Simulation Session: Evaluation of Six Simulated Patients with Different Shock Syndromes
Source: MedEdPORTAL. 2017 Jun 7;13:10591. doi: 10.15766/mep_2374-8265.10591 (PMC6354717; doi:10.15766/mep_2374-8265.10591)

| Appendix E: MedEdPORTAL Simulation Case  SIMULATION CASE TITLE: Multi-Patient Simulation Session: Evaluation of Six Simulated Patients with Different Shock Syndromes.  AUTHORS: Richard Lammers, MD, Philip Pazderka, MD, Maria Sheakley, PhD. | |
| --- | --- |
| PATIENT NAME: Mark Header  PATIENT AGE: 40  CHIEF COMPLAINT: Fall 20 feet from a ladder | |
|  | |
| Brief narrative description of case | This patient is a 40-year-old male who fell 20 feet from a ladder one hour prior to arrival. Witnesses report loss of consciousness for an unknown length of time. Prehospital providers placed the patient in cervical spine immobilization. The patient complains of head and neck pain. He is too confused to provide a reliable history. Student teams have been informed that they are members of a shock response team, and have eight minutes to evaluate the patient, record key clinical findings in a chart, view test results, and attempt a therapeutic intervention. |
| Primary Learning Objectives | By the end of this simulation session, the learner will be able to:   1. Assign roles to each team member to maximize team efficiency. 2. Evaluate the patient and record key clinical and diagnostic findings. 3. Initiate at least one therapeutic intervention. 4. Classify the type of shock based on data collected during the clinical encounters. 5. Identify the etiology of shock, or make a presumptive diagnosis. 6. Predict cardiac output, central venous pressure, and systemic vascular resistance. 7. Explain the physiologic and pharmacologic effects of the chosen therapy. |
| Critical Actions | 1. Assign roles to each team member at the beginning of the case. 2. Utilize the shock evaluation matrix to complete a focused history and physical exam. 3. Identify clinical findings consistent with spinal cord injury, including mechanism of injury, bradycardia and hypotension, cervical spine tenderness, and abnormal sensory and motor exam in upper and lower extremities. 4. Determine that the patient is in neurogenic shock. 5. Maintain cervical spine immobilization. 6. Deliver a fluid bolus of normal saline. |
| Learner Preparation | To prepare for this event, students should complete the following pre-reading assignments:   1. The clinical and hemodynamic characteristics of each of the classes of shock (See Critical Care Emergency Medicine. Section XI: Special Considerations; Chapter 46: Classification of Shock). 2. Winters, ME, DeBlieux P, Marcolinie EG, et al. *Emergency Department Resuscitation of the Critically Ill*. American College of Emergency Physicians (publisher), Dallas; 2011; Chapter 1: The Patient with Undifferentiated Shock, pp. 1-4. |

| INITIAL PRESENTATION | | | |
| --- | --- | --- | --- |
| Initial vital signs | Temp: 36.5^o^ C  Pulse: 56 /minute  Blood pressure: 85/50 mm Hg  Respirations: 16/minute  Oxygen saturation: 95%  Mean Arterial Pressure (MAP): 62 mm Hg | | |
| Overall Appearance | There is a middle-age, adult male wearing street clothes, in a cervical collar and lying supine on a backboard. He is awake, but confused. A pulse oximeter probe has been placed on a finger, and cardiac electrodes are properly placed. The vital signs monitor has been turned on. The patient is *not* receiving oxygen. Peripheral IV access has been established. The same array of treatment options for all cases in this exercise are visible on a cart, including vasopressors, an antihistamine, an antiarrhythmic, calcium and calcium channel blocker, and steroid drugs; IV fluids and blood products; airway equipment; a defibrillator; an 18-gauge angiocath needle; and a glucose measurement device. | | |
| Actors and roles in the room at case start | A nurse at the bedside introduces the patient, hands an ED Triage Note to the team, and awaits instructions. During the scenario, the nurse provides further scripted information, diagnostic test results, and requested equipment. The nurse will describe physical findings that cannot be portrayed by the mannequin while staying in role. The nurse performs only those interventions requested by the learners. The nurse troubleshoots equipment and attempts to mitigate simulation artifacts that interfere with the case. The nurse receives instructions through an earpiece from an instructor in the Control Room, as needed. A simulation technician or other health care provider with basic medical knowledge (eg. EMT level) and who is familiar with the capabilities of the mannequin can play this role.  Nurse’s Initial Script:   - I’m nurse ___________________ . - This patient was just brought in by paramedics. - Here is my note and your chart. - It looks like he’s got a closed head injury. He can’t give much information. He just keeps repeating himself. - But, I’m more concerned about his blood pressure—it’s fairly low for brain trauma. - I placed an IV already.   A faculty instructor is present in the Control Room. This person serves as the voice of the patient, operates the computer by triggering manual changes as scripted, guides the nurse/actor by direct-talk two-way radio, and terminates the scenario at eight minutes. The faculty instructor observes the performance of the team, provides feedback, and facilitates the debriefing/discussion session. | | |
| HPI | Information in ED Triage Note:  Patient name: Mark Header  Demographics: 40 y/o; male  ED arrival information: EMS  Chief complaint: Fall from ladder, height of 20 feet  Significant history/details: Struck hard surface outside; current outside temperature 55^o^F; loss of consciousness for unknown length of time; all history from paramedics.  Allergies: unknown  Home medications: unknown  Medical history: unknown  Surgical history: unknown  Social history: unknown  Safety screen (Feels safe at home?): unknown  Family comments: none  Vital signs:  T: 36.5^o^C  P: 56/min  BP: 85/50  R: 16/min  O_2_ sat: 95%  Nurse’s Evaluation: Still in c-collar; confused but cooperative; not able to provide much information because of amnesia; contusion on forehead; skin temp feels normal; not shivering  Treatment initiated: IV line  Information volunteered by patient: Presenting symptoms (head and neck pain);  Information provided by patient, if requested:  Too confused to provide a reliable history, and amnestic for the injury. Repeats statements (perseveration). Sensation to touch and pain intact in head and neck, decreased in lateral aspect of arms, absent in medial arms, below the elbows, and absent in trunk and lower extremities. | | |
| Past Medical/Surgical History | Medications | Allergies | Family History |
| unknown | unknown | unknown | unknown |
| Physical Examination | | | |
| General | *All findings are normal, except as described below:*  awake, confused, cooperative, in no respiratory distress | | |
| HEENT | forehead ecchymosis; normal pupil exam; moist oral mucous membranes | | |
| Neck | obscured by cervical collar; no jugular venous distention; trachea midline; tenderness in midline posteriorly, over mid-cervical spinous processes | | |
| Lungs | normal respiratory pattern; clear | | |
| Cardiovascular | bradycardia | | |
| Abdomen | non-tender; rectal tone present but weak; no blood | | |
| Neurological | *Mental status:* disoriented only to time; confused; repeats statements (perseveration); no recollection of injury  *Cranial nerves:* intact, including pupils  *Motor exam:* *Lower extremities:* no movement; *Upper extremities:* grade 0/5 hand grasp, finger abduction, and elbow extension; grade 1/5 wrist extension and elbow flexion; grade 2/5 shoulder abduction  *Sensory exam:* sensation intact in head and neck; decreased sensation in lateral aspect of arms; absent sensation in medial arms, below the elbows, trunk, and lower extremities; diminished sensation around anal orifice  *Deep tendon reflexes:* absent  *Cerebellar:* unable to test | | |
| Skin | dry; normal turgor and color | | |
| GU | no priapism | | |
| Psychiatric | mood & affect normal; cognition normal | | |

Diagnostic studies that are provided immediately, if ordered:

Complete blood count Normal Ranges:

White blood cells: 12.0 x 10^9^ cells/mcL (3.5-10.5 x 10^9^ cells/mcL)

Hemoglobin: 14.1 g/dL (13.5-17.5 g/dL)

Hematocrit: 41.5% (38.8-50%)

Platelets: 300,000 x10^3^ mcL (150-450 x10^3^ mcL)

Basic metabolic panel Normal Ranges:

Na (sodium): 145 mEq/L (135-144 mEq/L)

K (potassium): 3.5 mEq/L (3.7-5.2 mEq/L)

Cl (chloride): 99 mEq/L (97-108 mEq/L)

CO2 (bicarbonate): 30 mEq/L (22-29 mEq/L)

BUN (blood urea nitrogen): 15 mg/dL (7-20 mg/dL)

Cr (creatinine): 1.0 mg/dL (0.8-1.4 mg/dL)

Glucose: 100 mg/dL (64-128 mg/dL)

Ca (calcium) 8.8 mg/dL (8.5-10.6 mg/dL)

Lactic acid Normal Ranges:

Lactic acid: 2.5 (mEq/L) (0.5-2.2 mEq/L)

Radiology report

Chest Radiograph (Plain Film; AP view): Normal; poor inspiration


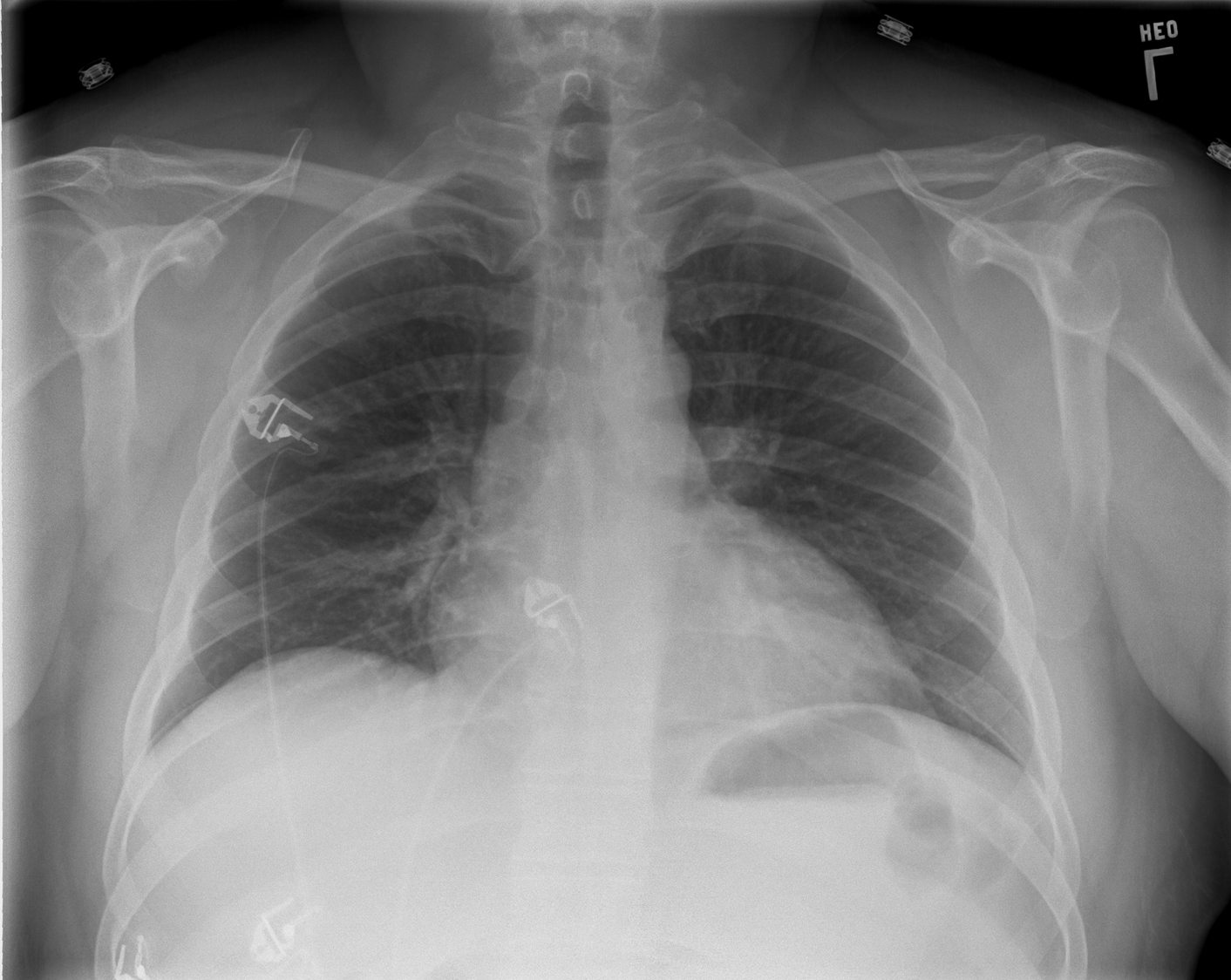


*Image from the collection of Richard Lammers, MD*

Cervical Spine Radiograph (Plain Film; Cross-table lateral view) - image not available:

Radiologist report: “Fracture of the arch of C5, with anterior subluxation of C5 on C6; small avulsion fracture of the anterior/inferior aspect of the vertebral body of C5, with associated widening of the pre-vertebral soft tissues. CT scan of cervical spine is recommended.”

12-lead ECG:


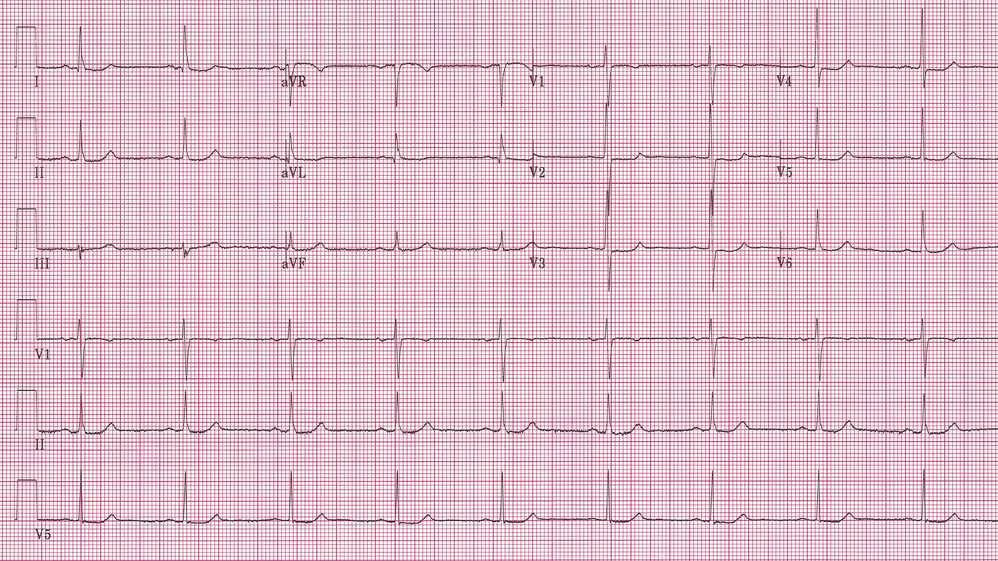


*Image from the collection of Richard Lammers, MD*

Rapid Ultrasound for Shock and Hypotension (RUSH) Examination Protocol

Subcostal Cardiac View: No pericardial effusion is present.

Apical 4 Chamber Cardiac View: Right ventricular size is normal.

Parasternal Long Axis Cardiac View: LV function is normal.

Inferior Vena Cava View: Inferior vena cava in the short axis measures 2.0 cm. Respiratory variability is > 50%. Conclusion: volume responsive.

Right & Left Upper Quadrant Views: No intraperitoneal fluid present.

Pelvic View: No intraperitoneal fluid present in sagittal or transverse planes.

Abdominal Aorta View: Aortic diameter is < 3 cm.

Thoracic View: No evidence of pneumothorax on right or left side.

| INSTRUCTOR NOTES - CHANGES AND CASE BRANCH POINTS | | |
| --- | --- | --- |
| Intervention / Time point | Change in Case | Additional Information |
| The same array of treatment options for all cases in this simulation exercise are visible on a cart. There is generally one best treatment option for each case. | | |
| *Dopamine IV drip* | *BP increases 10/10 mmHg*  *Pulse increases 20 bpm* | *Greater affinity for beta receptors than alpha receptors (B1 > a1 and B2)* |
| *Norepinephrine IV drip* | *BP increases 10/10 mmHg*  *Pulse increases 20 bpm* | *Greater affinity for beta receptors than alpha receptors(B1 > a1 and B2)* |
| *Epinephrine IV drip* | *BP increases 10/10 mmHg*  *Pulse remains unchanged* | *Greater affinity for beta receptors than alpha receptors (B1 > a1 and B2)* |
| *Epinephrine IM 0.3mg* | *BP increases 10/10 mmHg*  *Pulse increases 20 bpm* | *Greater affinity for beta receptors than alpha receptors (B1 > a1 and B2)* |
| *Phenylephrine IV drip* | *BP increases 10/10 mmHg*  *Pulse increases 20 bpm* | Appropriate treatment  *Acts on alpha-1 receptors, no effect on beta receptors.* |
| *Benadryl 50mg IV* | *BP remains unchanged*  *Pulse remains unchanged* |  |
| *Normal Saline Bolus 1 Liter IV* | BP increases 10/10 mm Hg | Appropriate treatment |
| *Needle thoracostomy* | Respirations increase 10/min  Oxygen saturation decreases 15% |  |
| *Synchronized cardioversion at 200J* | Respirations increase 5/min | No change in rhythm |

Ideal Scenario Flow

*Provide a detailed narrative description of the way this case should flow if participants perform in the ideal fashion.*

*The learners assign team roles and approach the patient. They should immediately review the bedside monitors and recognize that the patient is hypotensive and relatively bradycardic. They should provide supplemental oxygen and order an IV fluid bolus. After attempting to obtain history and completing a detailed trauma and neurologic examination, the learners identify signs of closed head injury and spinal cord injury, including neck tenderness and neurologic deficits at the C5 level. They maintain cervical spine immobilization throughout the entire evaluation. Fluid resuscitation provides adequate hemodynamic stabilization. The team can conclude that the patient is in neurogenic shock but should plan to evaluate the patient further for potential sources of internal hemorrhage. A trauma surgeon should be notified and diagnostic studies ordered.*

Anticipated Management Mistakes

*Provide a list of management errors or difficulties that are commonly encountered when using this simulation case.*

*Incomplete clinical skills: Depending on students’ level of knowledge of the nervous system and prior clinical skills training and basic science courses, the team may not know how to perform a thorough neurologic exam. However, a basic exam will reveal paralysis and sensory loss.*

*Incomplete exam: Medical student teams often fail to examine each system when confronted with time pressures.*

*Incorrect clinical reasoning: Some teams incorrectly assume that the cause of shock is due to blood loss from intracranial hemorrhage, which may lead them to withhold IV fluids.*

Completed shock evaluation matrix for Mark Header:
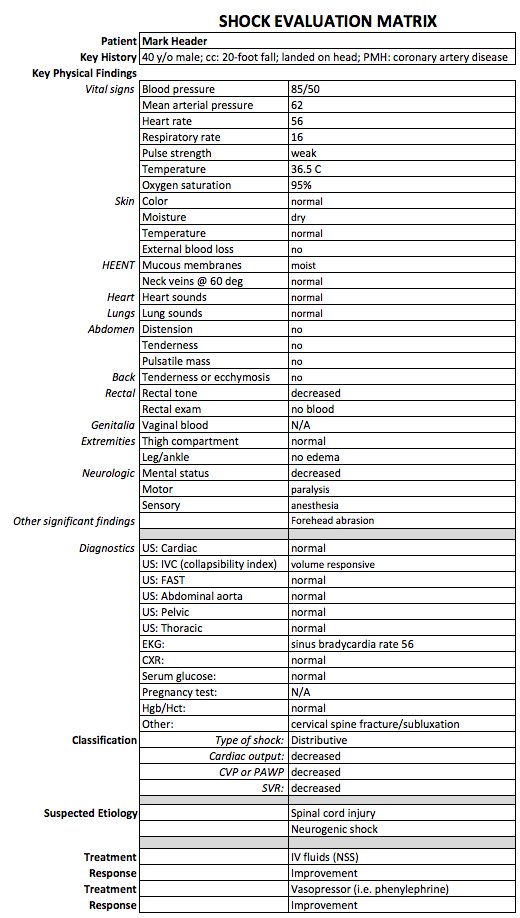

Supplement: Supplementary file 1 — A. Prereading Assignment.docx B. Patient 1 Scenario.docx C. Patient 2 Scenario.docx D. Patient 3 Scenario.docx E. Patient 4 Scenario.docx F. Patient 5 Scenario.docx G. Patient 6 Scenario.docx H. Preformatted Evaluation Matrix.xlsx I. Completed Evaluation Matrix.xlsx J. Survey Instrument.docx [file mep-13-10591-s001.zip › E._Patient_4_Scenario.docx]
