# Supplementary material for: A Multipatient Simulation Session: Evaluation of Six Simulated Patients with Different Shock Syndromes
Source: MedEdPORTAL. 2017 Jun 7;13:10591. doi: 10.15766/mep_2374-8265.10591 (PMC6354717; doi:10.15766/mep_2374-8265.10591)

| Appendix F: MedEdPORTAL Simulation Case  SIMULATION CASE TITLE: Multi-Patient Simulation Session: Evaluation of Six Simulated Patients with Different Shock Syndromes.  AUTHORS: Richard Lammers, MD, Philip Pazderka, MD, Maria Sheakley, PhD. | |
| --- | --- |
| PATIENT NAME: May Swoon  PATIENT AGE: 60  CHIEF COMPLAINT: Syncope | |
|  | |
| Brief narrative description of case | This patient is a 60-year-old female who had a syncopal episode after standing up to go to the bathroom in the Radiology Department. Patient completed a CT scan with contrast for right lower quadrant abdominal pain 10 minutes ago. Initial symptoms included “feeling funny” and mild shortness of breath. Currently, she reports a scattered rash, generalized itching, and worsening nausea. She did not strike her head when she fell, and she reports no new pain, but only the original pain in her abdomen. Student teams have been informed that they are members of a shock response team, and have eight minutes to evaluate the patient, record key clinical findings in a chart, view test results, and attempt a therapeutic intervention. |
| Primary Learning Objectives | By the end of this simulation session, the learner will be able to:   1. Assign roles to each team member to maximize team efficiency. 2. Evaluate the patient and record key clinical and diagnostic findings. 3. Initiate at least one therapeutic intervention. 4. Classify the type of shock based on data collected during the clinical encounters. 5. Identify the etiology of shock, or make a presumptive diagnosis. 6. Predict cardiac output, central venous pressure, and systemic vascular resistance. 7. Explain the physiologic and pharmacologic effects of the chosen therapy. |
| Critical Actions | 1. Assign roles to each team member before entering the patient room, ensuring that someone is assigned the role of scribe and another serves as team leader. 2. Utilize the shock evaluation matrix to complete a focused history and physical exam. 3. Identify clinical findings consistent with an anaphylactic reaction, including hypotension, tachycardia, rash, wheezing, low jugular venous pulse (JVP). 4. Determine that the patient is in distributive shock due to anaphylaxis. 5. Order epinephrine 0.3mg IM as treatment. |
| Learner Preparation | To prepare for this event, students should complete the following pre-reading assignments:   1. The clinical and hemodynamic characteristics of each of the classes of shock (See Critical Care Emergency Medicine. Section XI: Special Considerations; Chapter 46: Classification of Shock). 2. Winters, ME, BeBlieux P, Marcolinie EG, et al. *Emergency Department Resuscitation of the Critically Ill*. American College of Emergency Physicians (publisher), Dallas; 2011; Chapter 1: The Patient with Undifferentiated Shock, pp. 1-4. |

| INITIAL PRESENTATION | | | |
| --- | --- | --- | --- |
| Initial vital signs | Temp: 37.5^o^ C  Pulse: 130 /minute  Blood pressure: 80/40 mmHg  Respirations: 34/minute  Oxygen saturation: 93%  Mean Arterial Pressure (MAP): 53 mm Hg | | |
| Overall Appearance | When the learners enter the room, there is an adult female who is wearing a hospital gown, sitting at a 60-degree angle. She is mildly anxious, in mild respiratory distress. A pulse oximeter probe has been placed on a finger, and a nasal cannula in place; oxygen flow at 2 L/min. The vital signs monitor has been turned on. Peripheral IV access has been established. The same array of treatment options for all cases in this exercise are visible on a cart, including vasopressors, an antihistamine, an antiarrhythmic, calcium and calcium channel blocker, and steroid drugs; IV fluids and blood products; airway equipment; a defibrillator; an 18-gauge angiocath needle; and a glucose measurement device. | | |
| Actors and roles in the room at case start | A nurse at the bedside introduces the patient, hands an ED Triage Note to the team (see below in HPI section), and awaits instructions. During the scenario, the nurse provides further scripted information, diagnostic test results, and requested equipment. The nurse will describe physical findings that cannot be portrayed by the mannequin while staying in role. The nurse performs only those interventions requested by the learners. The nurse troubleshoots equipment and attempts to mitigate simulation artifacts that interfere with the case. The nurse receives instructions through an earpiece from an instructor in the Control Room, as needed. A simulation technician or other health care provider with basic medical knowledge (eg. EMT level) and who is familiar with the capabilities of the mannequin can play this role. If the team fails to recognize the symptoms and signs of anaphylaxis after 4 minutes, the nurse can ask, “Do you think that the CT scan caused this?”  Nurse’s Introduction Script:   - Hello, I’m nurse __________. - This patient just collapsed and we called you, are you the Shock Team? - Her blood pressure is low and she has a rash. - Here is your chart, the patient has an IV already*.* - Just let me know what you want me to do.   A faculty instructor is present in the Control Room. This person serves as the voice of the patient, operates the computer by triggering manual changes as scripted, guides the nurse/actor by direct-talk two-way radio, and terminates the scenario at eight minutes. The faculty instructor observes the performance of the team, provides feedback, and facilitates the debriefing/discussion session. | | |
| HPI | 60-year-old female who had a syncopal episode after standing up to go to the bathroom in the Radiology Department. The patient completed a CT scan with IV contrast within 5-10 minutes of the event. Initial symptoms included “feeling funny” and mild shortness of breath. Currently, she reports a scattered rash, generalized itching, and worsening nausea. She did not strike her head when she fell, and she reports no new pain, but only the original pain in her abdomen.  Information in ED Triage Note:  Patient name: May Swoon  Demographics: 60 y/o; female  Study ordered: abdominal CT with IV and oral contrast  Service requesting study: Family Medicine in-Patient Service; Dr. Lotta Payne  Indication for study: RLQ abdominal pain; rule out appendicitis, mesenteric ischemia, diverticulitis, etc.  Allergies: aspirin  Home medications: albuteral  Medical history: asthma  Incident: After patient completed CT scan, she got off stretcher to go to the bathroom because of worsening nausea. States she “felt funny”, got short of breath, itchy, then dizzy. She fainted, but did not strike her head when she fell, and she reports no new pain, but only the original pain in her abdomen. IV line in place and still functional. Called Shock Team stat. Care transferred to Shock Team immediately upon their arrival.  Vital signs in Radiology Department: (initial)  P: 130/min  BP: 80/40  R: 34/min  O_2_ sat: 93%  Vital signs on Hospital Unit:  T: 37.5^o^ C  P: 85/min  BP: 130/80  R: 12/min  O_2_ sat: 99%  Information volunteered by patient: Presenting symptoms (dizzy, rash, abdominal pain);  Information provided by patient, if requested:  Feeling dizzy and short of breath. Experiencing abdominal pain in the right lower quadrant. Has a scattered rash, generalized itching, and worsening nausea. | | |
| Past Medical/Surgical History | Medications | Allergies | Family History |
| asthma,  nasal polyps | albuterol for asthma | aspirin | Negative |
| Physical Examination | | | |
| General | awake; mildly anxious, in mild respiratory distress | | |
| HEENT | mild swelling of lips; moist oral mucous membranes; no intraoral swelling or stridor | | |
| Neck | no jugular venous distention; trachea midline | | |
| Lungs | tachypnea; bilateral wheezes | | |
| Cardiovascular | tachycardia | | |
| Abdomen | moderate tenderness in the right lower quadrant; no pulsatile mass | | |
| Neurological | normal | | |
| Skin | dry; decreased turgor; flushed; urticaria on extremities, face | | |
| GU | normal | | |
| Psychiatric | awake; oriented to person, place, and time; anxious; cognition intact | | |

Diagnostic studies that will be provided immediately if ordered:

Complete blood count Normal Ranges:

White blood cells: 16.0 x 10^9^ cells/mcL (3.5-10.5 x 10^9^ cells/mcL)

Hemoglobin: 12.1 g/dL (13.5-17.5 g/dL)

Hematocrit: 36.3% (38.8-50%)

Platelets: 400,000 x10^3^ mcL (150-450 x10^3^ mcL)

Basic metabolic panel Normal Ranges:

Na (sodium): 143 mEq/L (135-144 mEq/L)

K (potassium): 4.0 mEq/L (3.7-5.2 mEq/L)

Cl (chloride): 99 mEq/L (97-108 mEq/L)

CO2 (bicarbonate): 25 mEq/L (22-29 mEq/L)

BUN (blood urea nitrogen): 20 mg/dL (7-20 mg/dL)

Cr (creatinine): 1.3 mg/dL (0.8-1.4 mg/dL)

Glucose: 110 mg/dL (64-128 mg/dL)

Ca (calcium) 9.0 mg/dL (8.5-10.6 mg/dL)

Lactic acid Normal Range:

Lactic acid: 3.0 (mEq/L) (0.5-2.2 mEq/L)

RADIOLOGY REPORT

Chest Radiograph (Plain Film; AP view): Normal


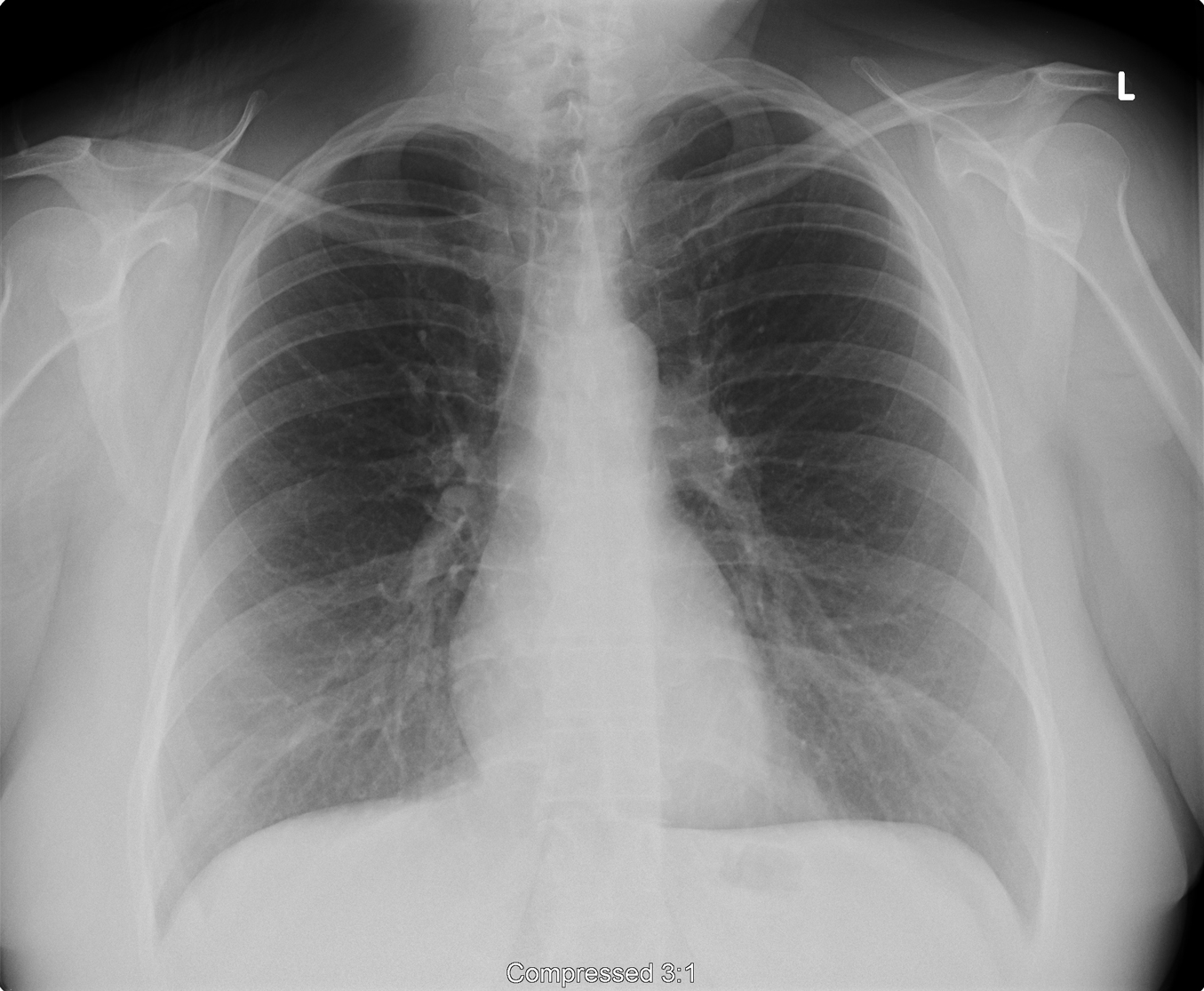


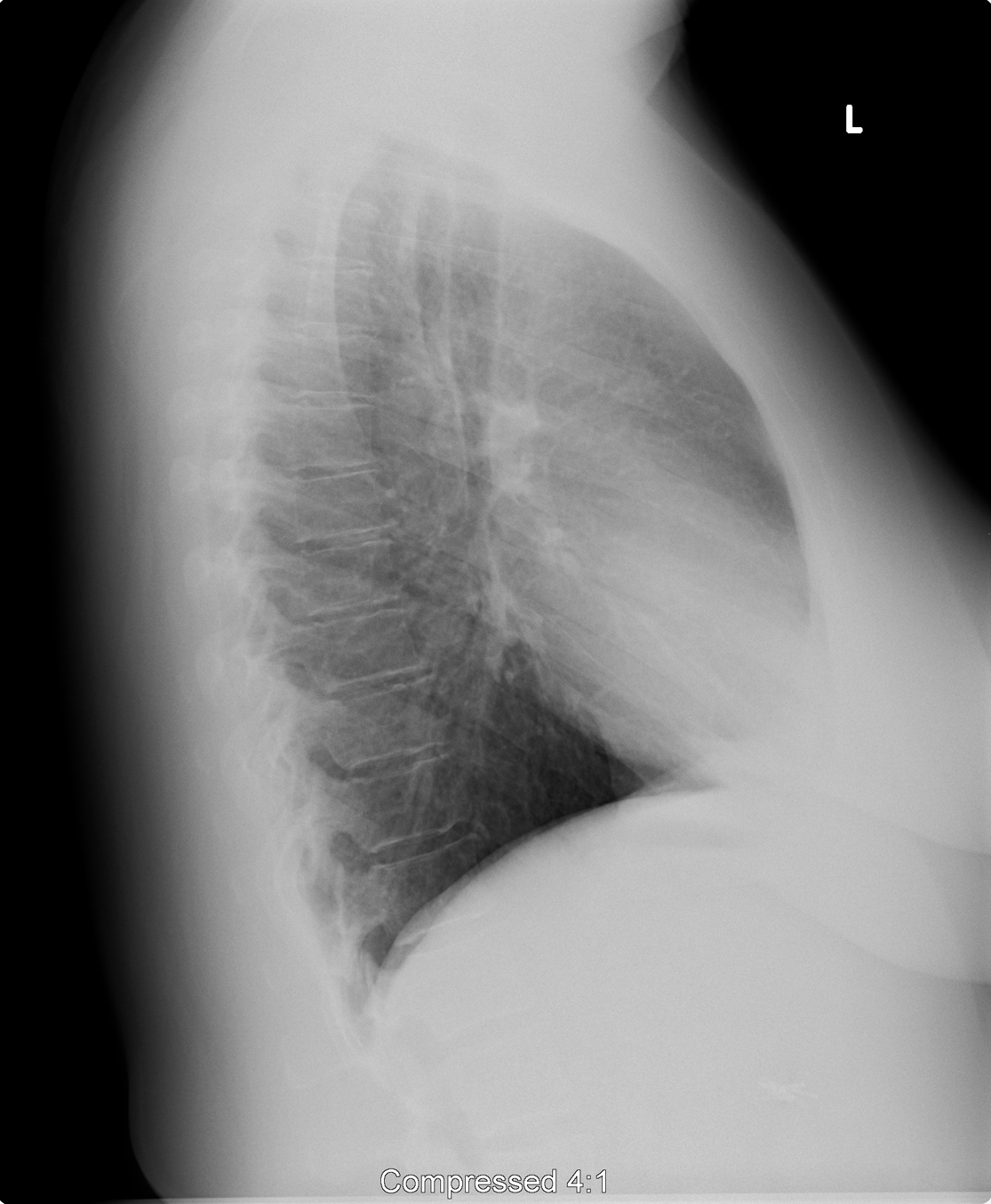


*Images from the collection of Richard Lammers, MD*

12-lead ECG


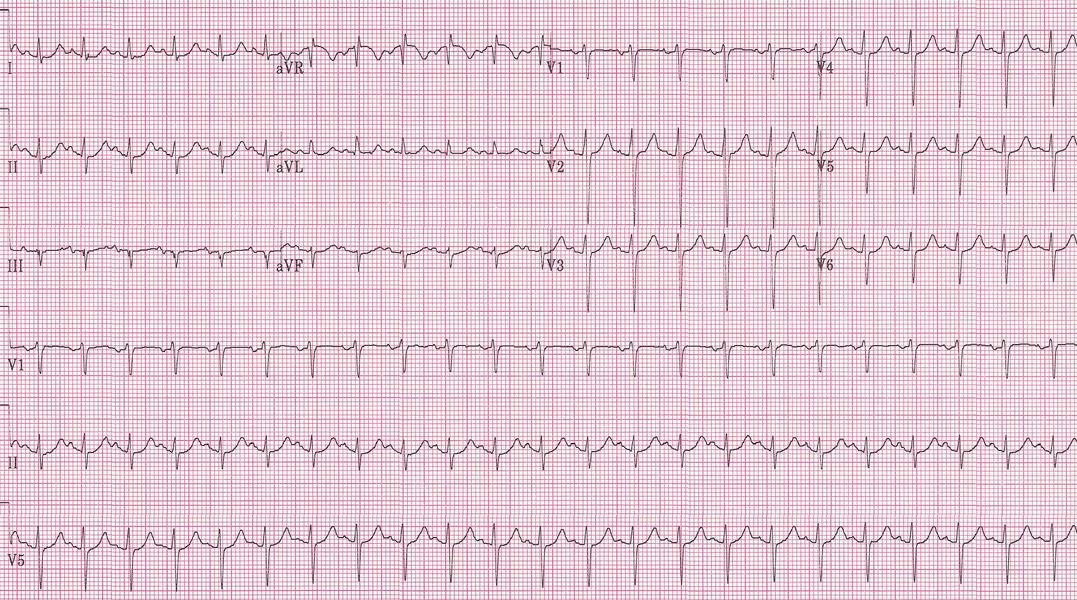


*Image from the collection of Richard Lammers, MD*

CT abdomen and pelvis:

Normal sized aorta, no perforation and no inflammatory changes of the bowel


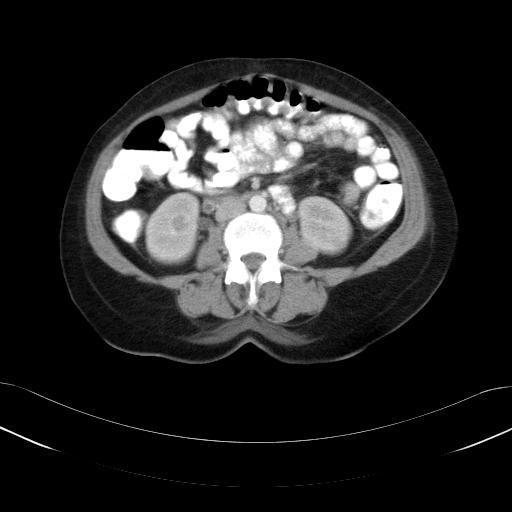


*Image from the collection of Richard Lammers, MD*

Rapid Ultrasound for Shock and Hypotension (RUSH) Examination Protocol

Subcostal Cardiac View: No pericardial effusion is present.

Apical 4 Chambered Cardiac View: Right ventricular size is normal.

Parasternal Long Axis Cardiac View: LV function is hyperdynamic.

Inferior Vena Cava View: Inferior vena cava in the short axis measures 2.0 cm. Respiratory variability is > 50%. Conclusion: volume responsive.

Right & Left Upper Quadrant Views: No intraperitoneal fluid present.

Pelvic View: No intraperitoneal fluid present in sagittal or transverse planes

Abdominal Aorta View: Aortic diameter is < 3 cm.

Thoracic View: No evidence of pneumothorax on right or left side.

| INSTRUCTOR NOTES - CHANGES AND CASE BRANCH POINTS | | |
| --- | --- | --- |
| Intervention / Time point | Change in Case | Additional Information |
| Array of treatment options for all cases in this simulation exercise are visible on a cart. There is generally one best treatment option for each case. | | |
| *Dopamine IV drip* | *BP increases 5/5 mmHg*  *Pulse increases 10 bpm* | *Greatest affinity for dopamine receptors. Greater affinity for beta receptors than alpha receptors (D1 > B1 and B2 > a1)* |
| *Norepinephrine IV drip* | *BP increases 10/10 mmHg*  *Pulse remains unchanged* | *Greater affinity for alpha receptors than beta receptors (a1 > B1))* |
| *Epinephrine IV drip* | *BP increases 10/10 mmHg*  *Pulse increases 20 bpm* | *Greater affinity for beta receptors than alpha receptors (B1 > a1 and B2)* |
| *Epinephrine IM 0.3mg* | *BP increases 10/10 mmHg*  *Pulse increases 20 bpm* | Appropriate treatment |
| *Phenylephrine IV drip* | *BP increases 10/10 mmHg*  *Pulse remains unchanged* |  |
| *Benadryl 50mg IV* | *BP remains unchanged*  *Pulse remains unchanged* | Appropriate treatment |
| *Normal Saline Bolus 1 Liter IV* | *BP increases 10/10 mmHg* | Appropriate treatment |
| *Needle thoracostomy* | Respirations increase 10 per minute, oxygen sat decreases 15% |  |
| *Synchronized cardioversion at 200J* | Respirations increase 5 per minute |  |

Ideal Scenario Flow

*Provide a detailed narrative description of the way this case should flow if participants perform in the ideal fashion.*

The learners enter the room to find an anxious 60-year-old female who had a syncopal episode after standing up to go to the bathroom in the Radiology Department. Patient completed a CT scan with contrast for right lower quadrant abdominal pain 10 minutes ago. Initial symptoms included “feeling funny” and mild shortness of breath. Currently, she reports a scattered rash, generalized itching, and worsening nausea. The patient is hypotensive and tachycardic. After completing a focused history and physical examination, the learners note that the patient’s mucus membranes are moist, pulses are weak, neck veins are not visible (at 60 degrees), an urticarial rash is present on the extremities and face, respiratory rate is elevated, and the patient is wheezing. The learners recognize that the patient is having an anaphylactic reaction, and order IM epinephrine. In response, the patient’s blood pressure increases and heart rate decreases. The learners will also order a normal saline bolus to aid in the treatment of hypotension, and IV Benadryl to treat the allergic reaction. A steroid such as solu-medrol should be used early to suppress the reaction once the effects of the faster-acting medications waned, but this should not be the only treatment.

Anticipated Management Mistakes

*Provide a list of management errors or difficulties that are commonly encountered when using this simulation case.*

*Difficulty predicting the patients cardiac output, central venous pressure, and systemic vascular resistance: During the debriefing session, we ask the teams to predict the patient’s CO, CVP, and SVR, based on the type of shock they are experiencing. This sometimes requires a facilitator to walk the groups through the thought process for this (i.e. MAP = CO x SVR) for the first few patients. The teams are typically able to figure this out on their own for the last 3-4 patients.*

*Failure to assign roles: Medical student teams may not assign roles or divide tasks among themselves, resulting in inefficiencies, or repeating some tasks while ignoring others.*

Complete shock evaluation matrix for May Swoon:


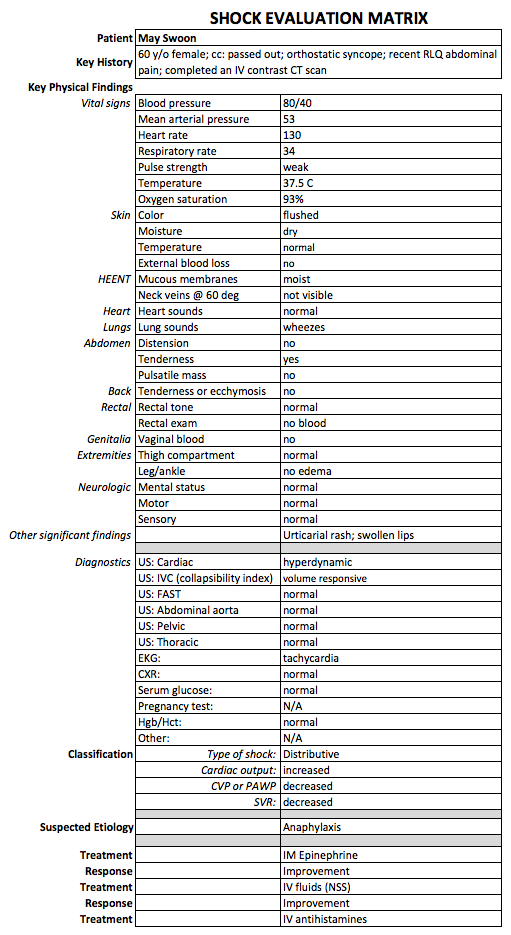

Supplement: Supplementary file 1 — A. Prereading Assignment.docx B. Patient 1 Scenario.docx C. Patient 2 Scenario.docx D. Patient 3 Scenario.docx E. Patient 4 Scenario.docx F. Patient 5 Scenario.docx G. Patient 6 Scenario.docx H. Preformatted Evaluation Matrix.xlsx I. Completed Evaluation Matrix.xlsx J. Survey Instrument.docx [file mep-13-10591-s001.zip › F._Patient_5_Scenario.docx]
