# Supplementary material for: A Multipatient Simulation Session: Evaluation of Six Simulated Patients with Different Shock Syndromes
Source: MedEdPORTAL. 2017 Jun 7;13:10591. doi: 10.15766/mep_2374-8265.10591 (PMC6354717; doi:10.15766/mep_2374-8265.10591)

| Appendix G: MedEdPORTAL Simulation Case  SIMULATION CASE TITLE: Multi-Patient Simulation Session: Evaluation of Six Simulated Patients with Different Shock Syndromes.  AUTHORS: Richard Lammers, MD, Philip Pazderka, MD, Maria Sheakley, PhD. | |
| --- | --- |
| PATIENT NAME: Victor Pector  PATIENT AGE: 64  CHIEF COMPLAINT: Chest pain and shortness of breath | |
|  | |
| Brief narrative description of case | This patient is a 64-year-old male admitted to the Internal Medicine Hospitalist Service today for cellulitis of his forearm. He has received one intravenous dose of antibiotics. He developed shortness of breath and chest pain suddenly while walking to the bathroom. Student teams have been informed that they are members of a shock response team, and have eight minutes to evaluate the patient, record key clinical findings in a chart, view test results, and attempt a therapeutic intervention. |
| Primary Learning Objectives | By the end of this simulation session, the learner will be able to:   1. Assign roles to each team member to maximize team efficiency. 2. Evaluate the patient and record key clinical and diagnostic findings. 3. Initiate at least one therapeutic intervention. 4. Classify the type of shock based on data collected during the clinical encounters. 5. Identify the etiology of shock, or make a presumptive diagnosis. 6. Predict cardiac output, central venous pressure, and systemic vascular resistance. 7. Explain the physiologic and pharmacologic effects of the chosen therapy. |
| Critical Actions*.* | 1. Assign roles to each team member before entering the patient room, ensuring that someone assumes the role of scribe and another serves as team leader. 2. Utilize the shock evaluation matrix to complete a focused history and physical exam. 3. Identify clinical findings consistent with a myocardial infarction, including dyspnea, hypotension, and tachycardia. 4. Determine that the patient is in cardiogenic shock. 5. Initiate norepinephrine treatment as a bridge to definitive therapy. |
| Learner Preparation | To prepare for this event, students should complete the following pre-reading assignments:   1. The clinical and hemodynamic characteristics of each of the classes of shock (See Critical Care Emergency Medicine. Section XI: Special Considerations; Chapter 46: Classification of Shock). 2. Winters, ME, BeBlieux P, Marcolinie EG, et al. *Emergency Department Resuscitation of the Critically Ill*. American College of Emergency Physicians (publisher), Dallas; 2011; Chapter 1: The Patient with Undifferentiated Shock, pp. 1-4. |

| INITIAL PRESENTATION | | | |
| --- | --- | --- | --- |
| Initial vital signs | Temp: 38.5^o^ C  Pulse: 90 /minute  Blood pressure: 60/40 mm Hg  Respirations: 34/minute  Oxygen saturation: 88%  Mean Arterial Pressure (MAP): 47 mm Hg | | |
| Overall Appearance | When the learners enter the room, there is a middle-aged, adult male who is wearing a hospital gown, sitting at a 60-degree angle, and is in moderate distress and diaphoretic. A pulse oximeter probe has been placed on a finger, and cardiac electrodes are properly placed. The vital signs monitor has been turned on. The patient is *not* receiving oxygen. Peripheral IV access has been established. The same array of treatment options for all cases in this exercise are visible on a cart, including vasopressors, an antihistamine, an antiarrhythmic, calcium and calcium channel blocker, and steroid drugs; IV fluids and blood products; airway equipment; a defibrillator; an 18-gauge angiocath needle; and a glucose measurement device. | | |
| Actors and roles in the room at case start | A nurse at the bedside introduces the patient, hands an intern’s progress report (see below in HPI section), and awaits instructions. During the scenario, the nurse provides further scripted information, diagnostic test results, and requested equipment. The nurse will describe physical findings that cannot be portrayed by the mannequin while staying in role. The nurse performs only those interventions requested by the learners. The nurse troubleshoots equipment and attempts to mitigate simulation artifacts that interfere with the case. The nurse receives instructions through an earpiece from an instructor in the Control Room, as needed. A simulation technician or other health care provider with basic medical knowledge (eg. EMT level) and who is familiar with the capabilities of the mannequin can play this role.  Nurse’s Initial Script:   - Hello, I’m nurse ___________________ . - Are you the Shock Team? - This patient is 64-years old male, and he has a history of coronary artery disease. - He was admitted to our unit for a cellulitis on his forearm. - Here is the intern’s progress report, and a chart for you to record your findings. - The intern gave him an aspirin and a nitroglycerin already. He’s turning over the case to you. - I placed an IV already. - Do you want me to give him something?   A faculty instructor is present in the Control Room. This person serves as the voice of the patient, operates the computer by triggering manual changes as scripted, guides the nurse/actor by direct-talk two-way radio, and terminates the scenario at eight minutes. The faculty instructor observes the performance of the team, provides feedback, and facilitates the debriefing/discussion session | | |
| HPI | PROGRESS REPORT  Internal Medicine (Hospitalist) Service  Patient name: Victor Pector  Demographics: 64 y/o male  Subjective: Hospitalized today for left forearm pain, redness, warmth, induration. Dx: cellulitis; Rx: received first dose of antibiotic 2 hours ago. Developed sudden onset of shortness of breath and chest pain while walking to the bathroom. Chest pain is central, described as “heaviness”, and 9/10 in severity. Duration of pain: 10 minutes. Admits to brief episodes of similar pain with exertion in the past two months. Significant PMH includes coronary artery disease, CABG 8 years ago. No allergies. Takes aspirin daily.  Objective: Agitated, in moderate respiratory distress; has difficulty speaking because of dyspnea; looks pale & diaphoretic—all new symptoms. Febrile; not tachycardic.  Current vital signs:  T: 38.5^o^ C  P: 90/min  BP: 60/40  R: 34/min  O_2_ sat: 88%  Assessment: New chest pain and shortness of breath, with hypotension. Consider allergic reaction, pulmonary embolus (source: left forearm venous system), cardiac event, pneumonia with developing sepsis, spontaneous pneumothorax, thoracic aortic dissection.  Plan: Begin fluid bolus with normal saline solution. Patient is more dyspneic when supine, so will keep at 60^o^ sitting position for now. Apply oxygen by nasal cannula, cardiac monitor. Consult Shock Team for further evaluation and treatment recommendations  Information volunteered by patient: Primary symptoms (chest pain and shortness of breath).  Information provided by patient, if requested:  Shortness of breath and severe chest pain and “heaviness” in the center of the chest. Pain is described as 9/10 in severity for the past 10 minutes. Has had brief episodes of similar pain with exertion in the past two months. Past medical history includes coronary artery disease. No allergies. Takes aspirin daily. | | |
| Past Medical/Surgical History | Medications | Allergies | Family History |
| Coronary artery disease | Aspirin | No known medical allergies | Not known |
| Physical Examination | | | |
| General | agitated; moderate respiratory distress; has difficulty speaking because of dyspnea | | |
| HEENT | moist oral mucous membranes; no intraoral swelling or stridor | | |
| Neck | jugular venous distention present at 60^o^ upright position; trachea midline | | |
| Lungs | tachypnea; rales bilaterally in all lung fields | | |
| Cardiovascular | normal rate; holosystolic, high-pitched blowing murmur, IV/VI | | |
| Abdomen | non-tender; no pulsatile mass | | |
| Neurological | normal; no focal findings | | |
| Skin | moist; cool; pale; normal turgor; 4 x 8 cm area redness & induration left forearm; no urticaria | | |
| GU | normal | | |
| Psychiatric | awake, though slightly slowed mentation; oriented to person, place, and time; mood normal; cognition intact | | |

Diagnostic studies that are provided immediately, if ordered:

Complete Blood Count Normal Ranges:

White blood cells: 14.0 x 10^9^ cells/mcL (3.5-10.5 x 10^9^ cells/mcL)

Hemoglobin: 16.1 g/dL (13.5-17.5 g/dL)

Hematocrit: 48.3% (38.8-50%)

Platelets: 410,000 x10^3^ mcL (150-450 x10^3^ mcL)

Basic Metabolic Panel Normal Ranges:

Na (sodium): 142 mEq/L (135-144 mEq/L)

K (potassium): 4.1 mEq/L (3.7-5.2 mEq/L)

Cl (chloride): 101 mEq/L (97-108 mEq/L)

CO2 (bicarbonate): 28 mEq/L (22-29 mEq/L)

BUN (blood urea nitrogen): 26 mg/dL (7-20 mg/dL)

Cr (creatinine): 1.1 mg/dL (0.8-1.4 mg/dL)

Glucose: 125 mg/dL (64-128 mg/dL)

Ca (calcium) 9.2 mg/dL (8.5-10.6 mg/dL)

Lactic Acid Normal Ranges:

Lactic acid: 5.0 (mEq/L) (0.5-2.2 mEq/L)

12-lead ECG:


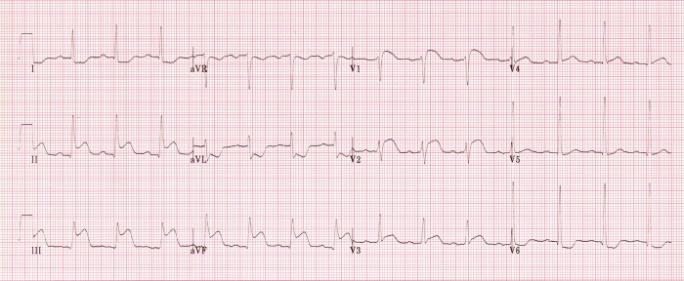


*Image from the collection of Richard Lammers, MD*

Radiology Report

Chest Radiograph (Plain Film; AP view):

“Heart: cardiac silhouette is slightly enlarged

Lungs: pulmonary venous congestion and mild edema; focal infiltrates in the right upper and right lower lobes could be consistent with pneumonia

Mediastinum & hilar structures: Aortic tortuosity and calcification; no overt aneurysmal dilatation

Bones: post-coronary artery bypass graft via midline sternotomy

Soft tissues: normal

Conclusion: 1) Radiographic features of congestive heart failure; 2) focal infiltrates in the right upper and right lower lobes could be consistent with pneumonia, in the proper clinical setting.”


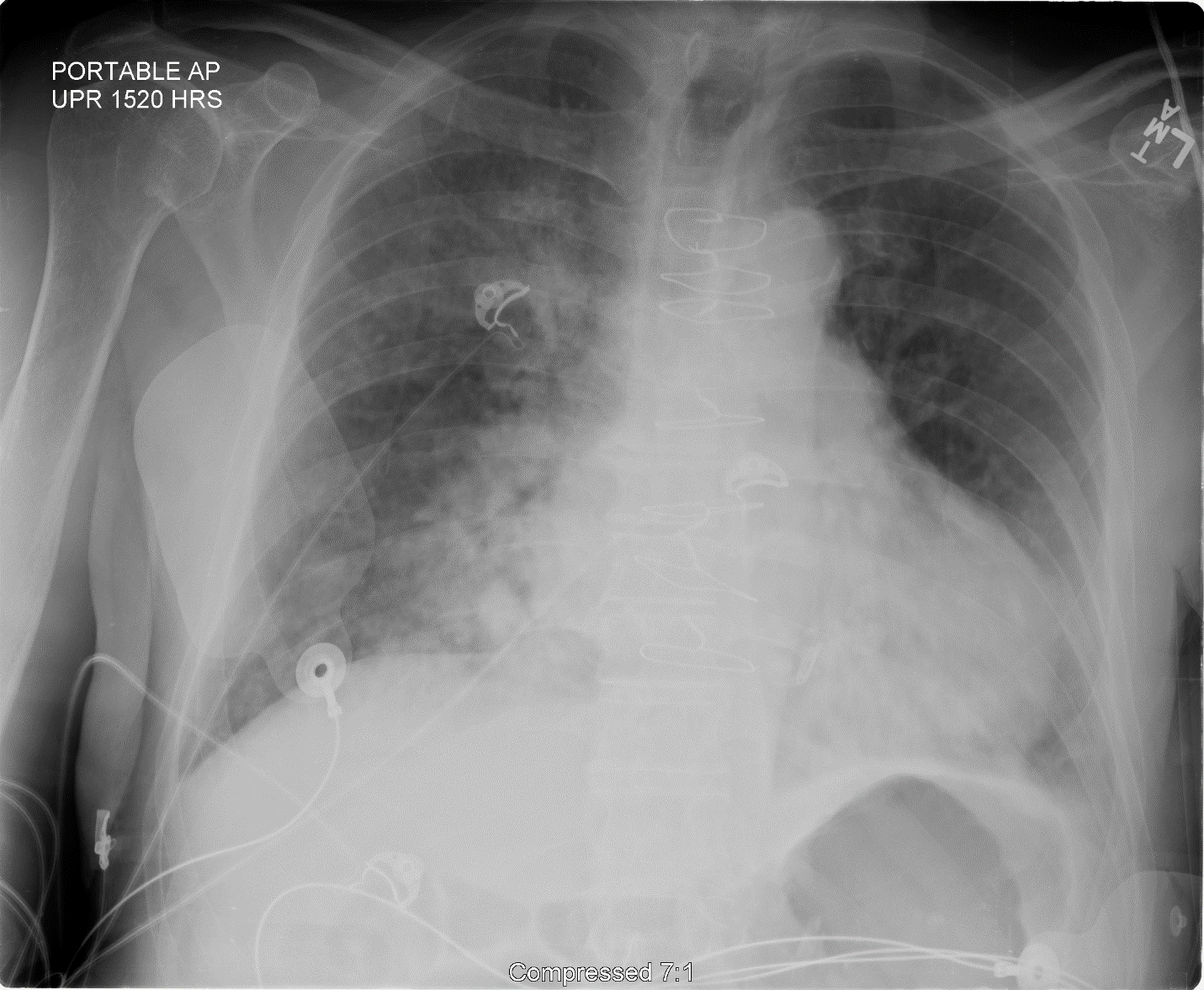


*Image from the collection of Richard Lammers, MD*

Rapid Ultrasound for Shock and Hypotension (RUSH) Examination Protocol

Subcostal Cardiac View: No pericardial effusion is present.

Apical 4 Chamber Cardiac View: Right ventricular size is dilated.

Parasternal Long Axis Cardiac View: LV function is hypodynamic.

Inferior Vena Cava View: Inferior vena cava in the short axis measures 2.5 cm. Respiratory variability is < 20%. Conclusion: volume unresponsive.

Right & Left Upper Quadrant Views: No intraperitoneal fluid present.

Pelvic View: No intraperitoneal fluid present in sagittal or transverse planes.

Abdominal Aorta View: Aortic diameter is < 3 cm.

Thoracic View: No evidence of pneumothorax on right or left side.

| INSTRUCTOR NOTES - CHANGES AND CASE BRANCH POINTS | | |
| --- | --- | --- |
| Intervention / Time point | Change in Case | Additional Information |
| *Dopamine IV drip* | *BP increases 5/5 mmHg*  *Pulse increases 10 bpm* | *Greatest affinity for dopamine receptors. Greater affinity for beta receptors than alpha receptors (D1 > B1 and B2 > a1)* |
| *Norepinephrine IV drip* | *BP increases 10/10 mmHg*  *Pulse remains unchanged* | Best treatment option for this case. Patient blood pressure will improve the most.  *Greater affinity for alpha receptors than beta receptors (a1 > B1))* |
| *Epinephrine IV drip* | *BP increases 10/10 mmHg*  *Pulse increases 20 bpm* | *Greater affinity for beta receptors than alpha receptors (B1 > a1 and B2)* |
| *Epinephrine IM 0.3mg* | *BP increases 10/10 mmHg*  *Pulse increases 20 bpm* | *Greater affinity for beta receptors than alpha receptors(B1 > a1 and B2)* |
| *Phenylephrine IV drip* | *BP increases 10/10 mmHg*  *Pulse remains unchanged* | *Acts on alpha-1 receptors, no effect on beta receptors.* |
| *Benadryl 50mg IV* | *BP remains unchanged*  *Pulse remains unchanged* |  |
| *Normal Saline Bolus 1 Liter IV* | *BP increases 10/10 mmHg* | *Patient will complain of increased dyspnea and develop rales. Respiratory rate will increase by 2 breaths, min. Oxygen saturation will decrease by 2%* |
| *Needle thoracostomy* | *Respirations increase 10 per minute, oxygen sat decreases 15%* |  |
| *Synchronized cardioversion at 200J* | *Respirations increase 5 per minute* | *No change in rhythm* |

Ideal Scenario Flow

*Provide a detailed narrative description of the way this case should flow if participants perform in the ideal fashion.*

*The learners enter the room to find a patient in moderate distress. They assign team roles and should immediately review the bedside monitors and recognize that the patient is hypoxic and hypotensive. The team leader assigns roles to each team member, if not done previously. Supplemental oxygen is provided and an IV fluid may be initiated. The nitroglycerin given to the patient by the intern has worsened the hypotension. After obtaining an abbreviated but appropriate history and completing a focused physical examination, the learners identify JVD and a holosytolic heart murmur. The patient’s distress gradually worsens during the evaluation and with IV fluids administration.* The learners recognize that the patient is having acute myocardial infarction with associated cardiogenic shock, and order IV norepinephrine. In response, the patient’s blood pressure increases.

Anticipated Management Mistakes

*Provide a list of management errors or difficulties that are commonly encountered when using this simulation case.*

*For example:*

*Difficulty predicting the patient’s cardiac output, central venous pressure, and systemic vascular resistance: During the debriefing session, the teams are asked to predict the patient’s CO, CVP, and SVR, based on the type of shock. A facilitator is sometimes required to walk the groups through this thought process (i.e. MAP = CO x SVR) for the first 1-2 patients. The teams are typically able to figure this out on their own for the remaining patients.*

*Diagnosis of underlying etiology: The teams are asked to determine the class of shock and underlying etiology of shock for each patient. 8/12 teams correctly diagnosed this as cardiogenic shock, but only 6/12 correctly diagnosed the underlying etiology of acute myocardial infarction. During the debriefing session, it was discovered that four of the groups did not order an ECG, and therefore missed the diagnosis of acute myocardial infarction and acute mitral regurgitation, which caused cardiogenic shock.*

*Initiating appropriate therapy: Cardiogenic shock secondary to myocardial infarction is a difficult case to manage. Initiating fluids can worsen the patient’s appearance and push them further into failure. Initiating vasopressors on this patient causing increasing myocardial oxygen demand, makes the student step back and think about appropriate therapy.*

Completed shock evaluation matrix for Victor Pector:


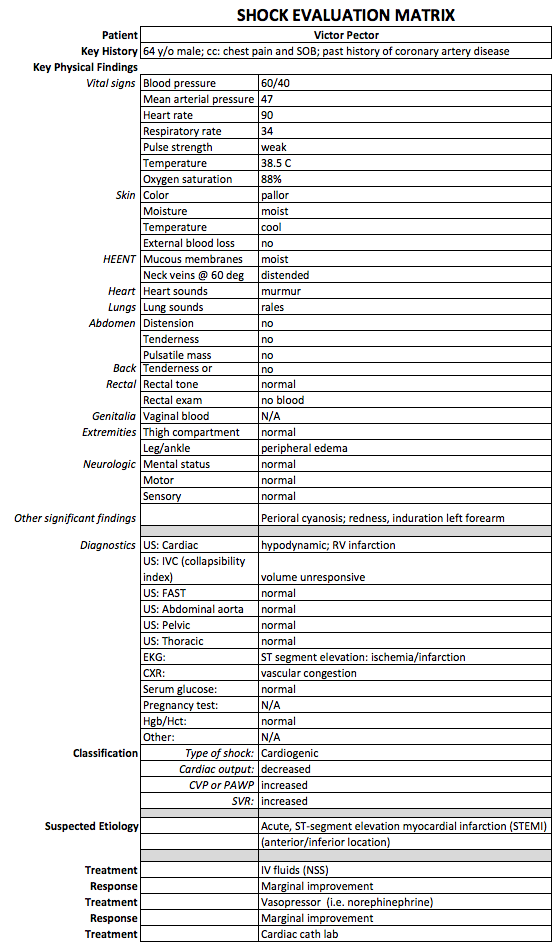

Supplement: Supplementary file 1 — A. Prereading Assignment.docx B. Patient 1 Scenario.docx C. Patient 2 Scenario.docx D. Patient 3 Scenario.docx E. Patient 4 Scenario.docx F. Patient 5 Scenario.docx G. Patient 6 Scenario.docx H. Preformatted Evaluation Matrix.xlsx I. Completed Evaluation Matrix.xlsx J. Survey Instrument.docx [file mep-13-10591-s001.zip › G_Patient_6_Scenario.docx]
