# Supplementary material for: A Multipatient Simulation Session: Evaluation of Six Simulated Patients with Different Shock Syndromes
Source: MedEdPORTAL. 2017 Jun 7;13:10591. doi: 10.15766/mep_2374-8265.10591 (PMC6354717; doi:10.15766/mep_2374-8265.10591)
Supplement: Supplementary file 1 — A. Prereading Assignment.docx B. Patient 1 Scenario.docx C. Patient 2 Scenario.docx D. Patient 3 Scenario.docx E. Patient 4 Scenario.docx F. Patient 5 Scenario.docx G. Patient 6 Scenario.docx H. Preformatted Evaluation Matrix.xlsx I. Completed Evaluation Matrix.xlsx J. Survey Instrument.docx [file mep-13-10591-s001.zip › J._Survey_Instrument.docx]

| **Instructions:** Rate how strongly you agree or disagree with each of the following statements about the Shock Simulation activity by circling the appropriate box, and provide written comments if desired. | | | | | | |
| --- | --- | --- | --- | --- | --- | --- |
|  | | Strongly Disagree | Disagree | Neutral | Agree | Strongly Agree |
| 1 | Pre-reading assignments prepared me for the shock simulation activity. | 1 | 2 | 3 | 4 | 5 |
| 2 | Briefing before the simulation was beneficial | 1 | 2 | 3 | 4 | 5 |
| 3 | Briefing before the simulation increased my confidence. | 1 | 2 | 3 | 4 | 5 |
| 4 | During the simulation, I had the opportunity to practice my clinical decision-making skills. | 1 | 2 | 3 | 4 | 5 |
| 5 | During the simulation, I had the opportunity to experience how time pressure can affect my clinical decision-making skills. | 1 | 2 | 3 | 4 | 5 |
| 6 | During the simulation, I had the opportunity to work as part of a healthcare team. | 1 | 2 | 3 | 4 | 5 |
| 7 | I am more confident in my ability to report information to my health care team. | 1 | 2 | 3 | 4 | 5 |
| 8 | I am more confident in my understanding of the pathophysiology of shock. | 1 | 2 | 3 | 4 | 5 |
| 9 | I am more confident in my ability to differentiate between different types of shock. | 1 | 2 | 3 | 4 | 5 |
| 10 | Debriefing contributed to my learning. | 1 | 2 | 3 | 4 | 5 |
| 11 | Debriefing was valuable in helping me select the appropriate treatments for different types of shock. | 1 | 2 | 3 | 4 | 5 |
| 12 | Debriefing provided adequate time to review the critical concepts related to shock. | 1 | 2 | 3 | 4 | 5 |
| 13 | Debriefing provided opportunities to self-reflect on my performance during the simulation. | 1 | 2 | 3 | 4 | 5 |
| Comments (optional): | | | | | | |
