# Supplementary material for: A Multipatient Simulation Session: Evaluation of Six Simulated Patients with Different Shock Syndromes
Source: MedEdPORTAL. 2017 Jun 7;13:10591. doi: 10.15766/mep_2374-8265.10591 (PMC6354717; doi:10.15766/mep_2374-8265.10591)
Supplement: Supplementary file 1 — A. Prereading Assignment.docx B. Patient 1 Scenario.docx C. Patient 2 Scenario.docx D. Patient 3 Scenario.docx E. Patient 4 Scenario.docx F. Patient 5 Scenario.docx G. Patient 6 Scenario.docx H. Preformatted Evaluation Matrix.xlsx I. Completed Evaluation Matrix.xlsx J. Survey Instrument.docx [file mep-13-10591-s001.zip › _Educational_Summary_Report_10591.pdf]

MedEdPORTAL®
The Journal of Teaching and Learning Resources

Original Publication

OPEN ACCESS

A Multipatient Simulation Session: Evaluation of Six Simulated Patients with Different Shock Syndromes

Richard Lammers, MD, Philip Pazderka, MD, Maria Sheakley, PhD*
*Corresponding author: maria.sheakley@med.wmich.edu

Citation: Lammers R, Pazderka P, Sheakley M. A multipatient simulation session: evaluation of six simulated patients with different shock syndromes. *MedEdPORTAL*. 2017;13:10591. https://doi.org/10.15766/mep_2374-8265.10591

Copyright: © 2017 Lammers et al. This is an open-access publication distributed under the terms of the Creative Commons Attribution-Share Alike license.

Abstract

Introduction: This multipatient simulation exercise was designed for second-year medical students to illustrate the four different categories of shock (hypovolemic, cardiogenic, obstructive, distributive) during a single simulation session. The comparative design of this simulation was intended to help students develop a conceptual framework for diagnosing and treating each type of shock. **Methods:** Students worked together in teams of five under specified time constraints to solve six simulated shock cases. The simulation exercise was implemented with a class of 60 second-year medical students. Teams collected key history and physical findings, established a working diagnosis, and administered treatment within an 8-minute window for each simulated patient. Following the simulations, a 90-minute facilitated discussion prompted students to compare and contrast the diagnoses and the basic management strategies for different types of shock using a preformatted shock evaluation matrix designed for the event. **Results:** The students applied basic science knowledge to the simulated clinical scenarios to diagnose the class and etiology of shock for each patient. The teams' ability to diagnose class of shock was better than their ability to determine the etiology. Students completed a voluntary evaluation of the educational exercise immediately following the simulation. **Discussion:** The unique, comparative design of this simulation provides educational value by exposing students to the various patterns of the four classes of shock in a single simulation session, presenting realistic clinical cases, and demonstrating the importance of teamwork in a time-pressured environment.

Keywords

Simulation, Teamwork, Shock, High-Fidelity Simulation, Editor's Choice, Multipatient Simulation

Appendices

A. Prereading Assignment .docx
B. Patient 1 Scenario.docx
C. Patient 2 Scenario.docx
D. Patient 3 Scenario.docx
E. Patient 4 Scenario.docx
F. Patient 5 Scenario.docx
G. Patient 6 Scenario.docx
H. Preformatted Evaluation Matrix.xlsx
I. Completed Evaluation Matrix.xlsx
J. Survey Instrument.docx

All appendices are peer reviewed as integral parts of the Original Publication.

Educational Objectives

By the end of this session, learners will be able to:

1. Assign roles to team members to maximize team efficiency.
2. Evaluate six simulated patients with different shock syndromes.
3. Record and report key clinical and diagnostic findings for each simulated clinical encounter.
4. Initiate at least one therapeutic intervention for each patient.
5. Classify the type of shock in each patient based on data collected during the clinical encounters.
6. Identify the etiology of shock, or make a presumptive diagnosis, for each patient.
7. Predict the cardiac output, central venous pressure, and systemic vascular resistance for each patient.
8. Explain the physiologic and pharmacologic effects of the chosen therapies for each patient.
9. Compare the clinical findings of each of the four classes of shock.

Introduction

A physiologic shock state is a life-threatening condition that occurs when perfusion is reduced to a level below which the body can adequately supply oxygen and nutrients to the tissue and organs. There are

four primary categories of shock, each having similarities and differences in patient presentation[1]; they include hypovolemic, cardiogenic, obstructive, and distributive.[2] Shock is a common condition, affecting up to one-third of patients in intensive care units[3]; thus, early exposure to it could be beneficial for medical students.

Undergraduate medical basic science curricula are rapidly adopting high-fidelity simulation as a modality to bridge the gap between the theory and practice of medicine,[4] and the topic of shock fits nicely into this model. The use of simulation reinforces the importance of teamwork and communication in patient management.[5] This allows students to learn about patient safety and to develop self-confidence and competence in basic clinical skills before being exposed to live patients.[6]

The Western Michigan University Homer Stryker M.D. School of Medicine has an integrated, organ system–based curriculum. This simulation occurred during the cardiovascular course, which is the first course of the second year. Prior to this event, the students completed the foundations courses (molecular, cell, genetics, and metabolics), as well as Immunology & Infectious Disease and Musculoskeletal & Dermatology courses. This simulation session was scheduled near the end of the cardiovascular course, after the students had learned all of the cardiovascular anatomy, physiology, imaging techniques (i.e., echocardiogram, ultrasound), and clinical tests (i.e., blood labs, electrocardiogram), as well as the full cardiac exam.

Numerous shock simulation cases have been published previously.[7-9] However, none have evaluated the four primary categories of shock in the same session. Our sequential case design allows students to experience each of the different shock presentations in quick succession and to compare and contrast the clinical findings in a single debriefing session. The purpose of this simulation event was to compare and contrast the diagnosis and basic management of different types of shock, to give students the opportunity to apply previously learned knowledge to simulated clinical scenarios, and to promote teamwork. This simulation experience is expected to link theory to practice and possibly improve students' confidence in evaluating shock cases in clerkships and residency.[10]

Methods

This study was a nonrandomized educational study for continuous quality improvement. It was designed to collect and evaluate student performance and survey data about a shock simulation activity in the second-year medical curriculum. This investigation was approved by the Institutional Review Board of Western Michigan University. Informed consent to participate in this medical simulation research study was accomplished through a verbal description of the protocol to groups of students and by subsequent voluntary completion of a survey questionnaire.

Sixty students from the class of 2019 completed this simulation activity as a normal part of the curriculum. The 60 students were divided into four groups of 15. The simulation session was 2.5 hours long and was run four times on different days to accommodate the entire class. No changes were made between each session. The 15 students scheduled each day were further divided into three groups of five students each. One week prior to the event, a prereading assignment was sent to the students in preparation for the simulation exercise (see Appendix A).

The students were informed that they would be members of a shock response team and would have to rapidly evaluate and treat six different patients suffering from various shock syndromes in hospital settings. On the day of the simulation, each team had 8 minutes to evaluate each simulated patient, record key clinical findings in a chart, view test results, and attempt at least one therapeutic intervention before moving to the next patient. There were six patients in total, each with a different underlying cause of shock. A sample schedule is provided below, and a diagram of the virtual hospital with the group rotation scheme is shown in Figure 1.

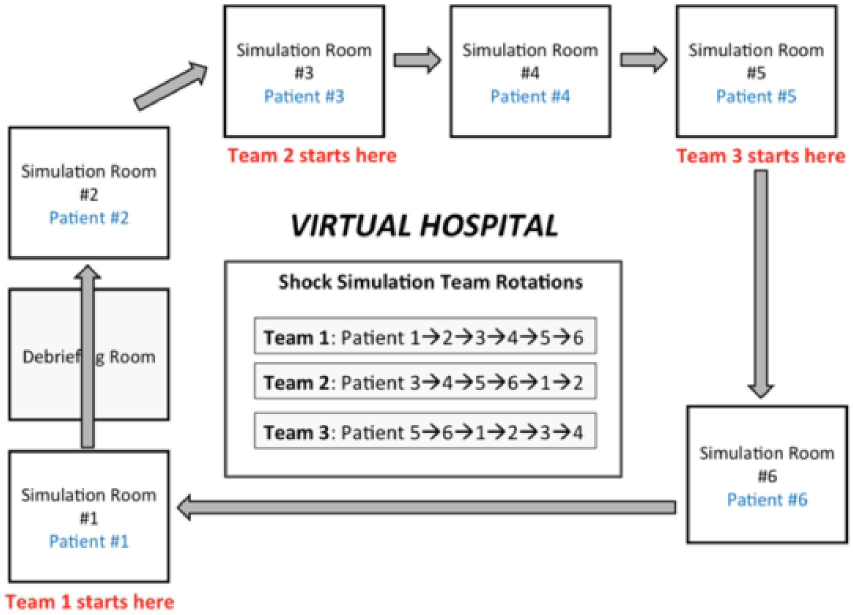

Figure 1. Diagram of virtual hospital and group rotation scheme.

- 3:00-3:05 p.m.: Brief introduction and explanation of the team rotation scheme.
- 3:05-3:13 p.m.: Patient 1—Jane Sweet (see simulation scenario, scripts, and programming in Appendix B).
- 3:13-3:21 p.m.: Patient 2—John Swift (see simulation scenario, scripts, and programming in Appendix C).
- 3:21-3:29 p.m.: Patient 3—Pat Roach (see simulation scenario, scripts, and programming in Appendix D).
- 3:29-3:37 p.m.: Patient 4—Mark Header (see simulation scenario, scripts, and programming in Appendix E).
- 3:37-3:45 p.m.: Patient 5—May Swoon (see simulation scenario, scripts, and programming in Appendix F).
- 3:45-3:53 p.m.: Patient 6—Victor Pector (see simulation scenario, scripts, and programming in Appendix G).
- 3:53-4:00 p.m.: Short break to transfer to debriefing classroom.
- 4:00-4:10 p.m.: Teams finalize patient charts, classify each type of shock, and make a presumptive diagnosis.
- 4:10-5:10 p.m.: Teams take turns reporting key findings for each patient (one case at a time) to the group and record the findings on a projected spreadsheet. The teams discuss the underlying physiologic causes of the major findings. There is 10 minutes for each case discussion.
- 5:10-5:30 p.m.: Wrap-up discussion comparing and contrasting major findings of each type of shock.

The teams were advised to assign roles (team leader, recorder, and support roles) to maximize efficiency and to rotate those role assignments as they moved from patient to patient. The simulation module was designed to give the groups just enough time to perform a focused history and physical in order to identify the cause and type of shock in each patient. In addition, a preformatted shock evaluation matrix (Appendix H) was provided to each group to use as a patient chart, to help them complete a focused exam, and to quickly organize the data collected for each patient. On the evaluation matrix, the groups could note key history findings, circle or write in key physical findings, and make notes about the

treatments administered and the patient's response to those treatments. For example, the group could circle *low, normal,* or *high* for the patient's blood pressure and record the actual blood pressure value, if measured. The preformatted evaluation matrix also contained a section for the groups to classify the class of shock syndrome and suspected etiology in each simulated patient based on the collected data.

Assessment

At the end of the session, the evaluation matrices were collected, and correct diagnoses of class and etiology of shock were tallied. The results are shown in aggregate in the Results section, below. In addition, the students voluntarily completed a 13-question qualitative evaluation of the session. The survey instrument utilized a 5-point Likert scale and an optional comments section. Responses are shown in aggregate in the Results section.

Equipment/Environment

Since this simulation includes six distinct cases, the equipment necessary to run each case (including mannequins, props, etc.) is detailed in each individual case appendix (Appendices B-G). In terms of available tests and treatments, the groups could order any laboratory, radiographic, or ultrasound tests they thought were appropriate. In this simulation design, time was compressed, so if the test results were available, they were given to the team immediately, though some tests may not have been available during the scenario. Nevertheless, the teams had access to enough information to make diagnosis and treatment decisions for each patient. The diagnostic tests available immediately upon request were as follows:

- Complete blood count (with normal ranges).
- Basic metabolic panel (with normal ranges).
- Lactic acid (with upper limits of normal).
- Cardiologist's report of electrocardiogram findings.
- Radiologist's report of chest plain film X-ray.
- Emergency physician's report of RUSH (Rapid Ultrasound for Shock and Hypotension) exam results.

In addition, a variety of simulated procedures, medications, and fluids/blood products were available for treatment:

- Procedures:
  - Trendelenberg position.
  - Left lateral decubitus position.
  - Needle thoracostomy.
  - Synchronized cardioversion—200 joules.
- Vasopressors:
  - Dopamine IV infusion.
  - Norepinephrine IV infusion.
  - Epinephrine IV infusion.
  - Epinephrine IM injection—0.3 mg.
  - Phenylephrine IV infusion.
- Other medications:
  - Glucagon IV bolus—5 mg.
  - Diphenhydramine (Benadryl) IV bolus—50 mg.
  - Solumedrol IV bolus—125 mg.
  - Calcium chloride IV bolus—1 ampule.
  - Diltiazem (calcium channel blocker) IV infusion—20 mg.
  - Amiodarone (anti-arrhythmic agent) IV bolus—300 mg.

- Broad-spectrum antibiotic IV infusion.
- Intravenous fluids:
  - Normal saline solution—1 liter.
  - Packed red blood cells—1 unit.

However, expert consultants (i.e., pharmacist or specialists) were not accessible.

Personnel

Since there were three groups of students rotating through the six scenarios, only three scenarios were being run simultaneously. This required three clinical faculty members to run the mannequins, alternating between scenarios. Each faculty member operated the mannequins from a control room, played the part of the patient in two scenarios (i.e., read the patient scripts), or controlled the prespecified responses of the mannequins as treatments were administered. Three simulation technicians as nurse actors were also required. They were instructed to follow scripts, provide information and cues included in those scripts, provide equipment, and deliver medications and fluids as requested by the student teams. The simulation technicians also alternated between scenarios as the student teams rotated, and they reset the rooms between scenarios. Finally, one additional simulation technician was present in the control room to time the scenarios, make sure the equipment was functioning properly, and assist as needed.

Debriefing

At the conclusion of the multipatient simulation exercise, all teams assembled in the classroom for a 90-minute debriefing session. At the start of the debrief session, the teams had 10 minutes to finalize the patient charts, classify each type of shock, and make a presumptive diagnosis, if not already done. The debriefing session was cofacilitated by a clinician, a basic science faculty member (physiologist), and a clinical pharmacologist. Teams took turns reporting their key findings to the group and recording them on the preformatted shock evaluation matrix form, which allowed the data for all six patients to be compared side by side (see the completed evaluation matrix in Appendix I). The evaluation form was projected on a large monitor for everyone to see during the debriefing session. Each team filled in the form for two patients while explaining the physiologic and pharmacologic effects of its chosen therapies; other teams were allowed to challenge its observations, decisions, or conclusions (10 minutes per case). The facilitators asked key questions about the causes of the findings being discussed during each case presentation to ensure understanding of the underlying physiology and pharmacology. As each case was presented, the findings were compared to previous cases, allowing students to recognize the various patterns of the four classes of shock (hypovolemic, cardiogenic, obstructive, and distributive). After all clinical data had been recorded on the preformatted evaluation matrix, students were asked to identify individual clinical findings or combinations of findings that helped to distinguish among the classes of shock in these patients. Examples of distinguishing findings included skin moisture, distension of neck veins, lung sounds, heart rate, physical signs of anaphylaxis, and signs of spinal cord injury. Data were entered into a Microsoft Excel spreadsheet and saved in a CSV file. Microsoft Excel was used for data analysis.

Results

Each of the 12 groups attempted to diagnose the class of shock and etiology of shock for each patient based on the patient history, physical exam, and test data they collected during the simulation. Figure 2 quantifies the aggregate number of correct diagnoses made by the 12 student groups for the class and etiology of shock for each of the six simulated cases. For the determination of class of shock, 100% of groups correctly diagnosed cases 1, 4, and 5; 92% of groups correctly diagnosed cases 2 and 3; and 67% of groups correctly diagnosed case 6. For the more difficult determination of etiology of shock, 83% of groups correctly diagnosed cases 3 and 5; 75% of groups correctly diagnosed cases 1 and 4; 50% of groups correctly diagnosed case 6; and 0% of groups correctly diagnosed case 2.

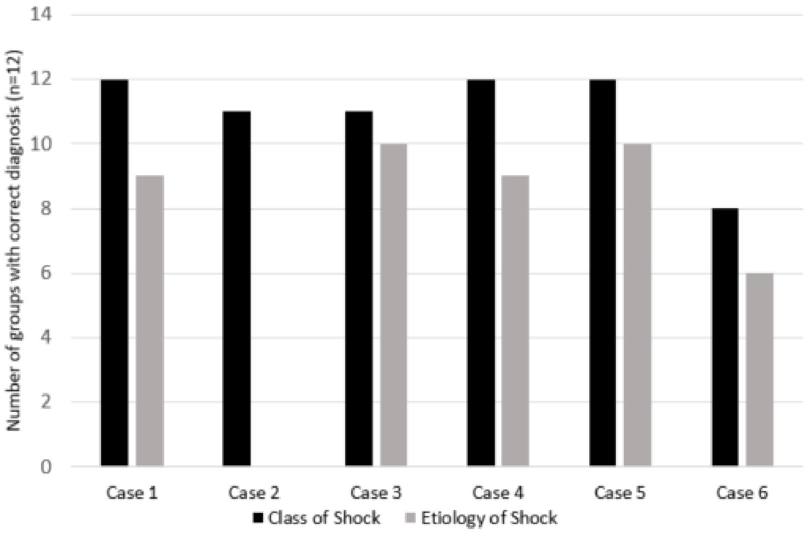

Figure 2. Number of correct diagnoses of class of shock and etiology of shock per case. Data are reported as aggregate number of groups with correct diagnosis (n = 12).

At the conclusion of the debriefing, students voluntarily completed a 13-question evaluation (Appendix J) of the session. The survey instrument, which was modified from an article by Leighton, Ravert, Mudra, and Macintosh,[11] utilized a 5-point Likert scale and an optional comments section. All 60 students voluntarily completed the survey. The results are shown in the Table.

Table. Student Responses to Survey Evaluating the Simulation Session (N = 60)

Survey Question	Strongly Disagree	Disagree	Neutral	Agree	Strongly Agree
1. Prereading assignments prepared me for the shock simulation activity.	0%	10%	30%	46%	15%
2. Briefing before the simulation was beneficial.	2%	13%	21%	44%	20%
3. Briefing before the simulation increased my confidence.	3%	13%	38%	23%	23%
4. During the simulation, I had the opportunity to practice my clinical decision-making skills.	0%	2%	5%	28%	66%
5. During the simulation, I had the opportunity to experience how time pressure can affect my clinical decision-making skills.	0%	2%	2%	21%	75%
6. During the simulation, I had the opportunity to work as part of a health care team.	0%	0%	7%	25%	69%
7. I am more confident in my ability to report information to my health care team.	2%	2%	13%	43%	41%
8. I am more confident in my understanding of the pathophysiology of shock.	2%	3%	10%	39%	46%
9. I am more confident in my ability to differentiate between different types of shock.	0%	5%	10%	25%	61%
10. Debriefing contributed to my learning.	0%	0%	8%	15%	77%
11. Debriefing was valuable in helping me select the appropriate treatments for different types of shock.	0%	2%	8%	21%	69%
12. Debriefing provided adequate time to review the critical concepts related to shock.	0%	2%	8%	39%	51%
13. Debriefing provided opportunities to self-reflect on my performance during the simulation.	0%	2%	10%	33%	56%

The optional student comments included the following:

- “Very valuable experience.”
- “Could have used more time on the first case to figure things out.”
- “It was a great simulation!”
- “Fantastic!”
- “It was great!”
- “This simulation was an important part of my understanding of shock.”
- “More knowledge on the topic before the simulation would have increased my confidence.”
- “5 min is short.”
- “Good learning opportunity.”
- “Thanks!”

- “This was well-planned and very fun!”
- “More time with each patient—10 minutes would have been great!”
- “Faculty did an excellent job teaching us how to make decisions in very emergent, time-limited clinical scenarios.”
- “It was enjoyable and educational.”

Discussion

Simulation added to traditional lecture-based education positively impacts knowledge acquisition, retention, and application of skills.[12] Chakravarthy and colleagues observed that presenting as much basic science education as possible in a clinical context increases knowledge retention.[13] Simulation does this effectively.

The purpose of this simulation activity was threefold: (1) to compare and contrast the diagnosis and basic management of different types of shock, (2) to give students the opportunity to apply previously learned basic science knowledge to simulated clinical scenarios, and (3) to promote teamwork. The comparative design of this session is unique among simulations on this topic, and it exposes students to the various patterns of all four classes of shock (hypovolemic, cardiogenic, obstructive, and distributive) in one simulation exercise. If an institution does not have the time, support, equipment, or resources to run all six patients simultaneously, two cases can be eliminated (as cardiogenic and distributive shock are represented by two cases each), or the cases can be used individually or in successive sessions. However, this may not produce as robust a discussion as the original design, since the comparative aspect would be diminished or lost. Nonetheless, it would allow students to diagnose and treat simulated patients with shock, apply basic science knowledge, and work in teams.

The development of this simulation was integrated and multidisciplinary. The patient cases and algorithms for each mannequin’s response to each potential treatment (see Computer Programming in Appendices B-G) were written by clinicians (Lammers and Pazderka) in order to portray accurate clinical patient presentations. A physiologist (Sheakley) reviewed the cases and enhanced integration of the basic science content. This intentional collaboration between clinicians and basic scientists supports curricular integration and well-rounded case development.

The groups performed well overall on the diagnosis of the class of shock for each case, with a correct diagnosis 91.6% of the time among the student groups. Thirty-three percent of groups misdiagnosed the class of shock for case 6. During the debriefing session, it was discovered that these groups did not order an electrocardiogram and therefore missed the diagnosis and classification of acute myocardial infarction and acute mitral regurgitation that caused cardiogenic shock.

The groups were less successful in determining the underlying etiology of shock for each case. The correct etiology was diagnosed 61.1% of the time. Surprisingly, none of the groups correctly diagnosed the etiology of case 2, which was a case of atrial fibrillation with rapid ventricular response causing cardiogenic shock. During the debriefing session, it became evident that the groups had difficulty identifying the atrial fibrillation on the electrocardiogram tracing since the heart rate was so rapid. This became a learning point in the debriefing sessions. In addition, 50% of groups incorrectly diagnosed the etiology of case 6, which was a case of ST segment elevation myocardial infarction causing cardiogenic shock. Four of six groups that missed this diagnosis did not order an electrocardiogram and therefore misdiagnosed both the class and etiology of shock.

All 60 students voluntarily completed an anonymous survey immediately following the debriefing. Questions 1-3 referred to the presimulation events, questions 4-6 referenced the simulation, questions 7-9 referenced the students’ confidence following the simulation, and questions 10-13 referred to the debriefing session. For questions 1 and 2, more than half of the students agreed or strongly agreed that the prereading and briefing before the simulation were beneficial (61% and 64%, respectively). However, for question 3, only 46% of students agreed that the briefing before the simulation increased their confidence. This is one area of the simulation we can improve upon by helping students to better

understand the purpose of the session as a whole and by alleviating anxiety. For questions 4-6, nearly all students responded positively that simulation provided an opportunity to practice clinical decision-making skills, experience how time pressure can affect decision-making skills, and experience working as part of a health care team (94%, 96%, and 94%, respectively). This was not surprising since simulation exercises required students to do all of these things during the session. For questions 7-9, a majority of students responded positively about increased confidence in their ability to report information to a health care team, ability to understand the pathophysiology of shock, and ability to differentiate between different types of shock (84%, 85%, and 86%, respectively). By working through the simulation cases and debriefing, students are better able to bridge the gap between the theory and practice of medicine, which helps to boost confidence. For questions 10-13, nearly all students agreed or strongly agreed that the debriefing contributed to their learning, helped them select appropriate treatments for shock, allowed time to review critical concepts related to shock, and provided an opportunity to self-reflect on their performance during the simulation (92%, 90%, 90%, and 89%, respectively). The debriefing session is where the learning framework is created and contextualization occurs, so it is important that students see the importance of this part of the session.

Although this simulation exercise was designed for medical students who have studied cardiovascular physiology and have learned how to perform a cardiac exam and interpret cardiac lab work and imaging, it could also be adapted for resident physician learners to reinforce fundamental pathophysiologic concepts. It is particularly useful for demonstrating the physiological mechanisms involved in shock.

The biggest challenge for this simulation is the extensive setup and planning required to successfully implement the multipatient session with limited faculty members and simulation technicians. We recommend that the amount of time allotted for each simulated patient be increased from 6 minutes to 8 minutes to allow students more time to capture the key findings from the patient history and physical and to administer treatments. The amount of time provided for discussion could also be increased by 20 minutes to include some discussion about effective teamwork. These time changes would increase the session by approximately 30 minutes, making it 2.5 hours long. These recommended time changes are represented in all documents for this case.

Richard Lammers, MD: Professor, Department of Emergency Medicine, Western Michigan University Homer Stryker MD School of Medicine; Assistant Dean of Simulation, Western Michigan University Homer Stryker MD School of Medicine; Department Director of Research, Western Michigan University Homer Stryker MD School of Medicine

Philip Pazderka, MD: Assistant Professor, Department of Emergency Medicine, Western Michigan University Homer Stryker MD School of Medicine; Program Director, Western Michigan University Homer Stryker MD School of Medicine

Maria Sheakley, PhD: Associate Professor, Department of Biomedical Sciences, Western Michigan University Homer Stryker MD School of Medicine

Disclosures
None to report.

Funding/Support
None to report.

Ethical Approval
This publication contains data obtained from human subjects and received ethical approval.

References

1. Weil MH, Shubin H. Proposed reclassification of shock states with special reference to distributive defects. *Adv Exp Med Biol.* 1971;23:13-23. https://doi.org/10.1007/978-1-4615-9014-9_3
2. Vincent J-L, De Backer D. Circulatory shock. *N Engl J Med.* 2013;369(18):1726-1734. https://doi.org/10.1056/NEJMra1208943
3. Sakr Y, Reinhart K, Vincent J-L, et al. Does dopamine administration in shock influence outcome? Results of the Sepsis

Occurrence in Acutely Ill Patients (SOAP) Study. *Crit Care Med.* 2006;34(3):589-597. https://doi.org/10.1097/01.CCM.0000201896.45809.E3

4. Weller JM. Simulation in undergraduate medical education: bridging the gap between theory and practice. *Med Educ.* 2004;38(1):32-38. https://doi.org/10.1111/j.1365-2923.2004.01739.x
5. Lateef F. Simulation-based learning: just like the real thing.*J Emerg Trauma Shock.* 2010;3(4):348-352. https://doi.org/10.4103/0974-2700.70743
6. Aboumatar HJ, Thompson D, Wu A, et al. Development and evaluation of a 3-day patient safety curriculum to advance knowledge, self-efficacy and system thinking among medical students. *BMJ Qual Saf.* 2012;21(5):416-422. https://doi.org/10.1136/bmjqs-2011-000463
7. Hegarty C, Binstadt E. Neurogenic shock simulation case. *MedEdPORTAL Publications*. 2011;7:9054. http://doi.org/10.15766/mep_2374-8265.9054
8. Ko P, Sarsfield M, Campoli J, Freeman R, Welch K. SHOCK! Three simulated case series for medical students. *MedEdPORTAL Publications*. 2014;10:9711. http://doi.org/10.15766/mep_2374-8265.9711
9. Paolo W. High fidelity simulation case: teaching septic shock with DIC.*MedEdPORTAL Publications*. 2014;10:9957. http://doi.org/10.15766/mep_2374-8265.9957
10. Muniandy RK, Nyein KK, Maujus F. Improving the self-confidence level of medical undergraduates during emergencies using high fidelity simulation. *Med J Malaysia.* 2015;70(5):300-302.
11. Leighton K, Ravert P, Mudra V, Macintosh C. Updating the Simulation Effectiveness Tool: item modifications and reevaluation of psychometric properties. *Nurs Educ Perspect.* 2015;36(5):317-323. https://doi.org/10.5480/15-1671
12. Kaddoura MA. Critical thinking skills of nursing students in lecture-based teaching and case-based learning.*Int J Scholarsh Teach Learn.* 2011;5(2):Article 20.
13. Chakravarthy B, ter Haar E, Bhat SS, McCoy CE, Denmark TK, Lotfipour S. Simulation in medical school education: review for emergency medicine. *West J Emerg Med.* 2011;12(4):461-466. https://doi.org/10.5811/westjem.2010.10.1909

Received: January 9, 2017 | **Accepted:** May 12, 2017 | **Published:** June 7, 2017
